# Hospital-Level Determinants of Mortality After Pancreas Surgery: A Global Infrastructure Analysis

**DOI:** 10.1097/SLA.0000000000007196

**Published:** 2026-09-09

**Authors:** Camila Hidalgo Salinas, Pascale Tinguely, Dimitri A. Raptis, Sterre van Herwijnen, Ammar A. Javed, Rafael Perera, Cristina R. Ferrone, Christopher L. Wolfgang, Giuseppe K. Fusai

**Affiliations:** 1 Department of Surgery, NYU Grossman School of Medicine, New York, NY; 2 Nuffield Department of Primary Care Health Sciences, Centre for Evidence-Based Medicine, University of Oxford, Radcliffe Observatory Quarter, Oxford, UK; 3 Department for Continuing Education, University of Oxford, Oxford, UK; 4 Department of HPB Surgery and Liver Transplant, Royal Free Hospital NHS Foundation Trust, London, UK; 5 Department of Visceral and Transplantation Surgery, University Hospital Zurich, University of Zurich, Zurich, Switzerland; 6 Organ Transplant Center of Excellence, King Faisal Specialist Hospital and Research Centre, Riyadh, Saudi Arabia; 7 Faculty of Medicine, Alfaisal University, Riyadh, Saudi Arabia; 8 Faculty of Medicine, Vrije Universiteit Amsterdam, Amsterdam, Netherlands; 9 Department of Surgery, Cedars-Sinai Medical Center, Los Angeles, CA; 10 Division of Surgery and Interventional Sciences, University College London, UK

**Keywords:** pancreas surgery, mortality, morbidity, pancreatic neoplasms, pancreatoduodenectomy, pancreatectomy

## Abstract

**Objective:**

To identify modifiable hospital-level determinants of mortality after pancreas surgery and assess their cumulative protective effects through the development of a Hospital Resource Index (HRI) on a global scale.

**Background:**

Ninety-day mortality rates after pancreas surgery vary globally (2%–10%); however, institutional factors affecting mortality remain poorly understood. Although caseload volume has driven centralization policies in many health care systems, it is not fully explanatory, and the impact of specific modifiable structural and process-related hospital factors remains unclear.

**Methods:**

We linked patient outcome data from the 2021 PancreasGroup.org global prospective cohort to hospital infrastructure data from the PancreasGroup.org global survey. Binary availability of 20 hospital-level factors was assessed. Factors significantly associated with mortality on univariable multilevel regression were combined into an additive HRI. Multivariable logistic regression evaluated associations between HRI and 90-day mortality.

**Results:**

Analyses included 2928 patients from 214 centers across 60 countries. Six hospital factors were significantly associated with lower mortality, including interventional radiology/endoscopy availability, structured nutrition support, evidence-based practice adoption, staff training, equipment maintenance, and favorable nurse-patient ratios. Each additional HRI point was associated with 12% lower mortality odds (OR: 0.88, 95% CI: 0.81–0.95, *P*=0.001), independent of caseload volume (OR: 0.73, *P*=0.014, optimal cutoff 55 cases per year), country’s development index, and patient and procedural characteristics. Patients treated at hospitals with all 6 HRI resources had 54% lower adjusted odds of 90-day mortality than those at hospitals with none (OR 0.46, 95% CI 0.30–0.70).

**Conclusions:**

The developed HRI showed cumulative protective effects on mortality after pancreas surgery, with volume remaining independently protective in the adjusted model, providing actionable targets for resource allocation to enhance pancreas surgery outcomes beyond centralization globally.

Ninety-day mortality after pancreas surgery varies widely, ranging from 2% at high-volume specialized centers to 10%.^1–3^ This has driven centralization policies in several health care systems, predicated on the consistent observation that higher-volume centers report lower mortality.^4–7^ However, volume itself is neither fully explanatory nor universally modifiable. The concentration of cases is also not realistic in many health systems due to geography, demographics, or resource constraints.^8–10^

Volume likely in part serves as a proxy for underlying structural and process-related factors such as staffing, infrastructure, protocols, and institutional culture, that directly influence whether patients survive complications. The failure-to-rescue concept supports this hypothesis, with high-volume centers achieving lower mortality not only by preventing complications, but by rescuing patients when complications occur.^11^ Identifying specific infrastructure elements that allow rescue could thus guide targeted investment to reduce surgical mortality risk in pancreas surgery. However, hospital-level determinants remain poorly characterized. Existing literature on surgical outcomes has focused predominantly on patient, disease, and surgical approach characteristics, with limited investigation of system-level factors.^12–15^ Where hospital characteristics were examined, studies largely relied on administrative databases from single countries, restricting generalizability and the range of institutional variation captured and limiting generalizability to diverse settings.^16^

Whether specific hospital-related structural or process factors have protective effects on mortality in pancreas surgery, and whether such effects are consistent across diverse health care settings, is unknown.

The objective of this study was to characterize factors that, through targeted investment, may offer a strategy to complement centralization or even an alternative in settings where centralization is not feasible, by linking hospital infrastructure data to patient outcomes from a global prospective cohort.^4^ The primary aim was to identify modifiable hospital-level determinants of 90-day mortality independent of patient or surgery-related characteristics and assess cumulative protective effects through the development of a Hospital Resource Index (HRI). The secondary aim was to define the minimal caseload threshold across all types of pancreatic surgery and assess the role of volume on mortality in the context of HRI in a global cohort.

## METHODS

### Study Design and Data

We performed a secondary analysis linking hospital infrastructure data from a global survey to patient outcomes from a global prospective cohort. The 2021 PancreasGroup.org collaborative snapshot study (NCT04652271) assessed 90-day morbidity and mortality after pancreas surgery across 354 centers in 67 countries.^3^ Data on the availability of hospital-related factors were extracted from a web-based survey on pancreas surgery, assessing hospital caseload volume and the respondents’ perception of factor importance, followed by a binary assessment of availability at their institution. This covered 20 hospital-level factors across 9 domains, including expertise, monitoring, infrastructure, responsiveness, protocols, collaboration, nutrition, workforce, and communication.^17^ The survey was distributed to all participating surgeons involved in the 2021 study^3^ from December 2023 to February 2024. Multiple responses from a single center were consolidated into a single institutional profile using modal responses to represent institutional consensus. Where responses were evenly split (available vs. absent), availability was coded as absent, given the uncertainty of true availability. The study was registered at the Royal Free London NHS Foundation Trust audit tracker and conducted in accordance with the STROBE and CROSS reporting guidelines.^18,19^

### Outcomes and Predictors

The primary endpoint was 90-day all-cause mortality, defined as death within 90 days of index pancreatic resection. Secondary endpoints included major complications, defined as Clavien-Dindo grade ≥3a.^20^

Hospital-level factors comprised 21 binary variables (present/absent) across 9 domains. The remaining 20 factors were assessed for association with mortality. From factors significantly associated with mortality, we developed an HRI using additive scoring, with each protective factor present contributing 1 point. To assess caseload volume exposure, we sought to validate recently published volume thresholds. Kotecha et al^4^ identified 45 pancreatoduodenectomies (PDs) per year as the threshold for lowest mortality using spline regression analysis.^4^ We tested this threshold in our cohort and additionally performed Youden index analysis to identify the optimal volume cutoff discriminating 90-day mortality risk across all pancreas resection types in our cohort.

### Statistical analysis

The intraclass correlation coefficient was calculated from a null multilevel model to quantify the proportion of mortality variance attributable to between-hospital differences. Associations between individual hospital factors and 90-day mortality were examined using univariable multilevel logistic regression with random intercepts for hospitals to account for patient clustering. Factors significantly associated with mortality (*P*<0.05) were included in the development of HRI.

We evaluated the association between HRI score and mortality using multivariable logistic regression, adjusting for patient age, ASA grade, Charlson comorbidity index, procedure type (left-sided pancreatectomy vs. PD/total pancreatectomy), indication (malignant vs. benign), and country Human Development Index (HDI) category. Cluster-robust standard errors were used to account for residual hospital-level correlation. A dose-response relationship with morality was tested by modeling HRI as a continuous variable. The association between HRI and major complications was also examined with the same covariate adjustment using logistic regression with cluster-robust standard errors. As a sensitivity analysis, a weighted composite HRI score was constructed using the absolute values of beta coefficients from univariate multilevel logistic regression models for each resource and compared the results to the additive HRI approach.

Pancreas surgery caseload volume by center was examined as a potential confounder. The a priori threshold of 45 cases per year was tested in patients undergoing PD and the overall data set. Youden index analysis was performed to identify the optimal cutoff in the current cohort. The independent contributions of volume and HRI were assessed by including the two in adjusted models. To examine whether infrastructure availability varied by setting, we assessed the relationship between HRI scores and country HDI category using a χ^2^ test for trend. Subgroup analyses then examined whether HRI effects on mortality varied by HDI category (low/middle, high, and very high). Effect modification was assessed through interaction terms.

Since all outcome variables and covariates included in regression analyses were mandatory fields in the data collection tools from both databases, complete case analysis was performed. Effect sizes are reported as odds ratios (OR) with 95% CIs. Basic model assumptions (absence of multicollinearity and linearity of the log-odds) were verified. Analyses were performed in Stata version 19 (StataCorp).

## RESULTS

From the centers involved in the initial PancreasGroup.org study,^3^ 60% completed the survey (214 centers across 60 countries), yielding a data set of 2928 patients undergoing pancreas surgery with linked hospital infrastructure data. The median age was 65 years (IQR: 56–73), 49% were female, 35% were ASA grade ≥3, and the median Charlson comorbidity index was 2 (IQR: 2–3). Operations comprised PD (65%, n=1890), left-sided pancreatectomy (27%, n=796), total pancreatectomy (8%, n=225), and central pancreatectomy or enucleation (1%, n=17). A total of 418 (14%) patients had concomitant venous and/or arterial resection. Malignant neoplasms were the indication for resection in 81% (n=2379), with pancreatic ductal adenocarcinoma (47%, n=1382) and ampullary carcinoma (10%, n=298) most common. Patients operated in very high, high, and low-to-middle HDI countries comprised 85% (n=2499), 11% (n=310), and 4% (n=119), respectively. Overall, 90-day mortality was 9% (n=276), and major complications occurred in 43% (n=1256). The intraclass correlation coefficient from the null model was 16%, indicating substantial between-hospital variation in mortality.

Of 20 hospital-level factors analyzed, 6 were statistically significantly associated with lower 90-day mortality on univariable multilevel analysis (Table 1). These included “interventional radiology and endoscopy on-site availability,” “structured nutritional support and counseling,” “adoption of emerging evidence-based best practice,” “systematic training of health care staff and nurses in the management of post-pancreatectomy complications,” “maintenance of equipment and facilities,” and “favorable nurse-patient ratios” (Supplementary Table 1, Supplemental Digital Content 2, https://links.lww.com/SLA/F1000). These 6 factors were combined into an additive HRI (range 0–6). The HRI median score was 5 (IQR: 4–6), with most patients (81%) being treated at hospitals with 4 or more resource factors, while 14% at hospitals with 2 or 3 factors and only 5% with 1 or none.

**TABLE 1:** Hospital-Related Factors Assessed in the Survey and 90-Day Mortality

Survey Question Number	Hospital Factor	Domain	Availability %	OR	95% CI	*P*
4b^*^	Interventional radiology/endoscopy on-site availability	Infrastructure	91	0.65	0.44–0.96	**0.030**
8a^*^	Structured nutrition support	Nutrition	90	0.66	0.46–0.95	**0.026**
7b^*^	Evidence adoption	Collaboration	81	0.67	0.50–0.89	**0.007**
9b^*^	Systematic staff training	Workforce	61	0.69	0.54–0.88	**0.003**
1c	Specialist HPB Interventional radiology/endoscopy availability	Expertise	92	0.70	0.46–1.03	0.070
4d^*^	Equipment maintenance	Infrastructure	84	0.70	0.51–0.95	**0.024**
1d	Academic hospital	Expertise	91	0.73	0.49–1.10	0.134
9a^*^	Favorable nurse-patient ratios	Workforce	64	0.75	0.59–0.97	**0.028**
6a	Guideline adherence	Protocols	76	0.77	0.58–1.02	0.066
6b	Discharge policies	Protocols	43	0.78	0.60–1.01	0.055
4c	Interventional radiology/endoscopy 24/7 availability	Infrastructure	79	0.80	0.60–1.07	0.138
10b	Shift handover efficiency	Communication	83	0.82	0.60–1.13	0.226
5a	Rapid response teams	Responsiveness	85	0.83	0.59–1.17	0.287
3a^†^	Advanced monitoring immediately postop	Monitoring	98	0.86	0.39–1.91	0.711
1b	Specialized HPB units/teams	Expertise	85	0.89	0.63–1.25	0.494
7a	MDT coordination	Collaboration	77	0.90	0.67–1.21	0.472
3b	Standardized monitoring protocols	Monitoring	88	0.98	0.67–1.43	0.917
4a	ICU availability/capacity	Infrastructure	92	0.98	0.62–1.53	0.915
10a	Team communication/coordination	Communication	84	1.01	0.72–1.44	0.936
1a^†^	HPB specialty training ≥2 surgeons	Expertise	98	1.01	0.39–2.57	0.989

Bold values are indicates factors included in HRI (*P* < 0.05).

Listed in order of decreasing odds ratios (ORs).

^*^ Factors included in HRI (*P*<0.05).

^†^ Factors with 98% availability, where associations may be limited by the ceiling effect.

Using the a priori mortality benefit threshold of 45 cases per year (threshold) in patients with PD (1890), centers exceeding the threshold (62% of patients) demonstrated significantly lower 90-day mortality compared with centers below this threshold (8.6% vs. 12.2%; OR: 0.68, 95% CI: 0.50–0.92, *P*=0.012). Youden index analysis across all pancreatic resections in our cohort identified 55 cases per year as the optimal volume threshold for discriminating 90-day mortality. High-volume centers (>55 cases per year, n=1584, 54% of patients) had significantly lower mortality than low-volume centers (8.2% vs. 10.9%; OR: 0.69, 95% CI: 0.53–0.89, *P*=0.004). This threshold was used in subsequent analyses.

In multivariable analysis adjusting for caseload volume, patient age, ASA grade, procedure type, indication, and country HDI category, each additional HRI point was associated with a 12% reduction in the odds of dying within 90 days of pancreas surgery (OR: 0.88, 95% CI: 0.81–0.95, *P*=0.001) (Fig. 1 and Supplementary Table 2, Supplemental Digital Content 2, https://links.lww.com/SLA/F1000). This dose-response relationship remained linear across the entire HRI range (χ^2^ test for linear trend: χ^2^=11.43, *P*=0.001) (Fig. 2). Compared with patients at hospitals with no available resources (HRI=0), those at hospitals with all 6 resources (HRI=6) had 54% lower odds of 90-day mortality (OR: 0.46, 95% CI: 0.30–0.70) (Supplementary Table 3, Supplemental Digital Content 2, https://links.lww.com/SLA/F1000). Higher hospital volume (OR: 0.73, 95% CI: 0.57–0.94, *P*=0.014) and left-sided pancreatic resections compared with all other resections (OR: 0.60, 95% CI: 0.43–0.82, *P*=0.014) were also associated with lower 90-day mortality. Sensitivity analysis using a weighted composite HRI score confirmed a statistically significant prediction for 90-day mortality (OR: 0.70 per unit weighted score, 95% CI: 0.57–0.86, *P*=0.001). The proportion of malignant indications did not differ significantly across HRI strata (86% at HRI 0–2, 81% at HRI 3–4, and 81% at HRI 5–6; *P*=0.116).

**FIGURE 1:**
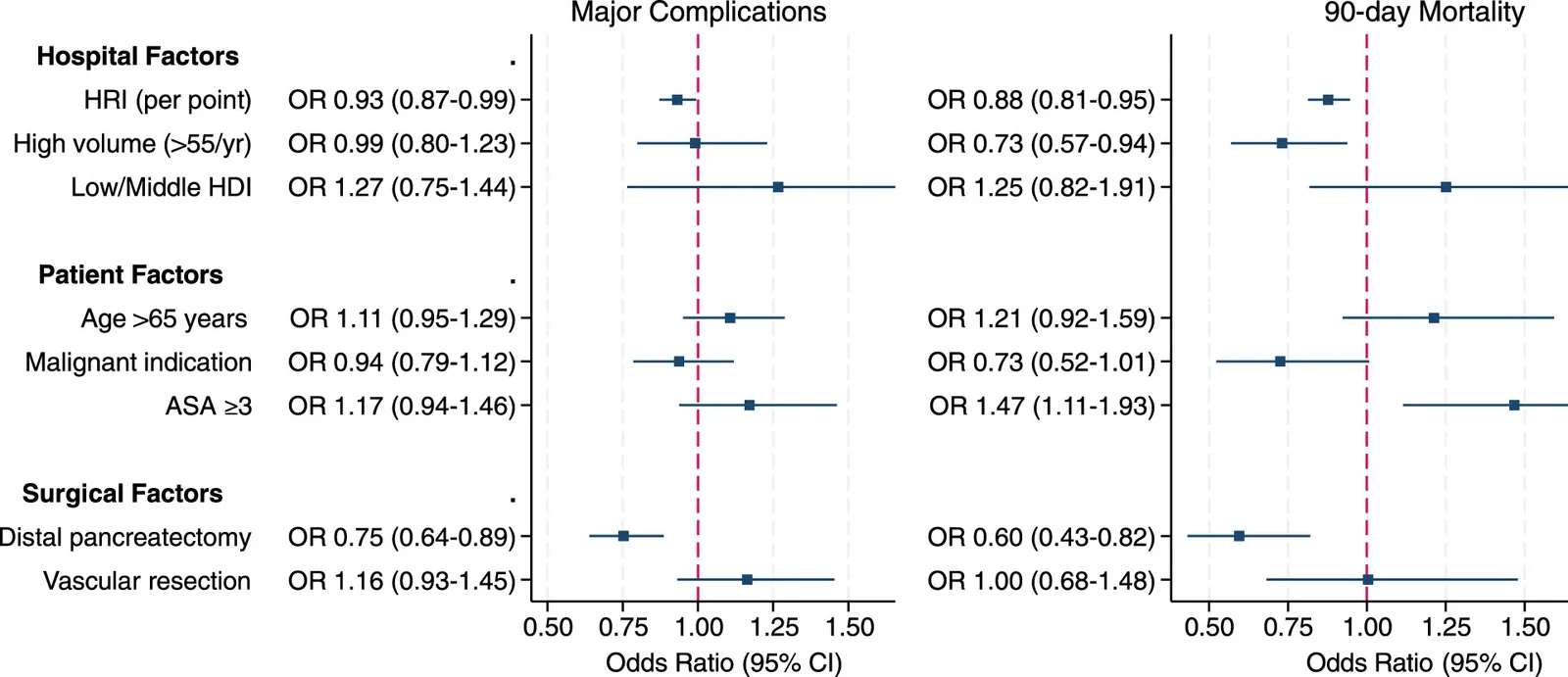
Multivariable predictors of major complications versus 90-day mortality. Multivariable logistic regression examining the association of Hospital Resource Index (HRI) as a continuous variable (score range 0–6) with major complications and 90-day mortality, adjusted for caseload volume, age, ASA score, malignancy, procedure type and vascular resection, and country human development index (HDI); reporting OR (95% CI). Each additional HRI point results in 12% reduction in the odds of dying within 90 days of pancreas surgery. Hospital Resource Index demonstrated protective effects on both complications (OR: 0.93, *P*=0.033) and mortality (OR: 0.88, *P*=0.001), with a stronger effect on mortality. Hospital volume showed no association with complications (OR: 0.99, *P*=0.932) but significantly reduced mortality (OR: 0.73, *P*=0.014). This pattern, whereby hospital factors reduce mortality without preventing complications, is consistent with the failure-to-rescue hypothesis: high-volume centers and those with greater infrastructure resources achieve lower mortality primarily through enhanced capacity to rescue patients when complications occur, rather than through complication prevention. The red vertical line marks the null value (OR=1). No evidence of problematic multicollinearity detected.

**FIGURE 2:**
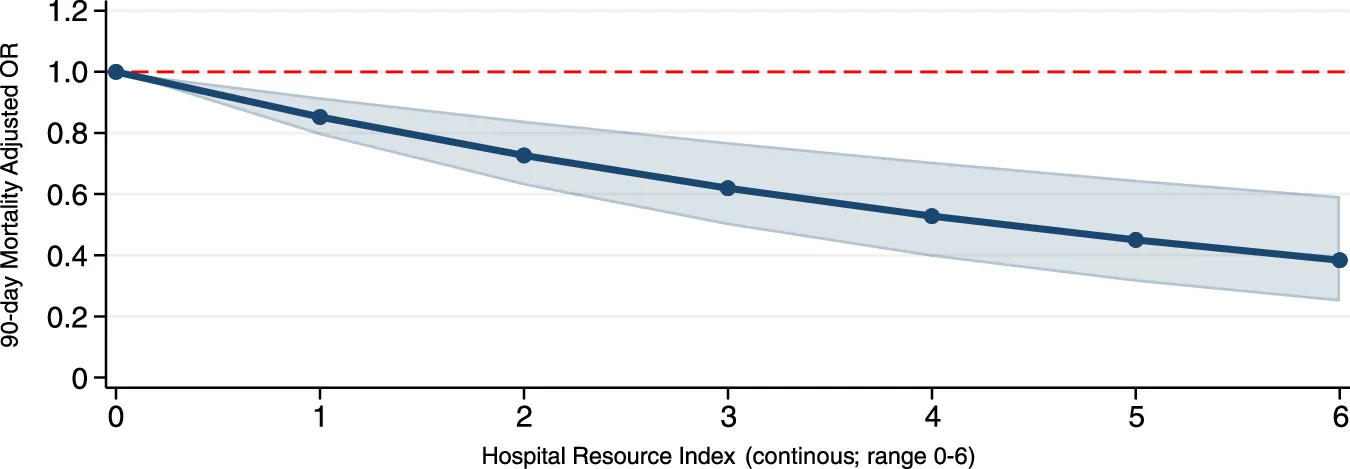
HRI dose-response relationship with 90-day mortality. Adjusted odds ratios for 90-day mortality per unit increase in HRI (0–6 scale). The solid line represents point estimates from multivariable logistic regression. The shaded band represents 95% CIs. The red dashed line indicates reference OR=1.0. Linear relationship confirmed by nonsignificant quadratic term test (*P*=0.43). Near-perfect correlation between the additive HRI (0–6 scale) and the beta coefficient weighted composite index confirmed (*r*=0.998, *P*<0.001).

Each additional HRI point was also associated with a 7% reduction in the odds of major complications (OR: 0.93, 95% CI: 0.87–0.99, *P*=0.033) in multivariable analysis (Fig. 1 and Supplementary Table 5, Supplemental Digital Content 2, https://links.lww.com/SLA/F1000). Left-sided pancreatic resection compared with all other resections was also independently associated with lower complication rates (OR: 0.75, 95% CI: 0.64–0.89, *P*=0.001). In contrast to the mortality analysis, hospital volume, ASA class, age, malignancy indication, vascular resection, and country HDI category were not significantly associated with major complications (all *P*>0.05).

Overall 90-day mortality was similar across HDI categories (9% in very high, 9% in high, and 12% in low-middle HDI countries; *P*=0.630). The protective effect of HRI on 90-day mortality was consistent across HDI categories; exploratory interaction adjusting for hospital volume and patient characteristics showed no significant interaction between HRI and HDI category (interaction OR: 0.96, 95% CI: 0.78–1.18, *P*=0.703) (Supplementary Table 4, Supplemental Digital Content 2, https://links.lww.com/SLA/F1000). In stratified analysis, hospital volume demonstrated a more pronounced protective effect in low-medium HDI countries (OR: 0.08 per category increase, 95% CI: 0.02–0.41, *P*=0.003) compared with high HDI countries (OR: 0.75, 95% CI: 0.58–0.97, *P*=0.029). HRI scores varied significantly by country HDI (Fig. 3), with each unit increase in HRI associated with a 32% reduction in the odds of being in a low-medium HDI country (OR: 0.68, 95% CI: 0.61–0.76, *P*<0.001).

**FIGURE 3:**
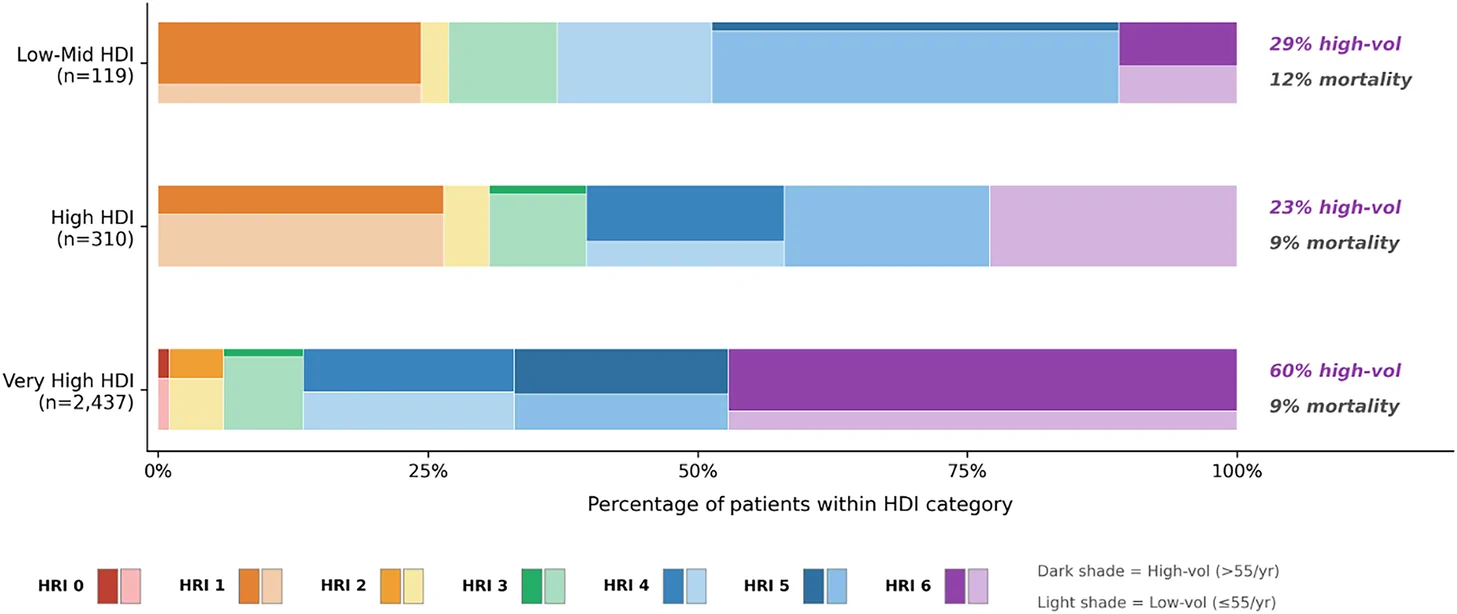
Hospital Resource Index distribution and high-volume center prevalence by country Human Development Index category. Stacked bar chart showing the percentage distribution of patients across Hospital Resource Index (HRI) scores (0–6), stratified by country HDI category. Within each HRI level, lighter shades represent patients at low-volume centers (≤55 cases per year) and darker shades represent high-volume centers (>55 cases per year). Overall 90-day mortality was 9% (234/2499) in very-high HDI, 9% (28/310) in high HDI, and 12% (14/119) in low-middle HDI countries. High-volume center prevalence varied substantially: 60% in very-high HDI, 23% in high HDI, and 29% in low-middle HDI countries.

## DISCUSSION

This study reports the first work showing that hospital-related structural factors have cumulative protective effects on mortality after pancreas surgery, beyond caseload volume and across varying health care settings worldwide in our cohort. Six modifiable hospital-level factors were identified that were independently associated with reduced 90-day mortality after pancreas surgery. The cumulative protective effect of these institutional factors on patient mortality was representatively captured by the HRI, with each additional resource reducing the mortality odds by 12% independent of patient characteristics, procedure complexity, caseload volume, and country HDI status. The consistency of this effect across health care settings in this cohort, combined with the known global inequality in resource distribution, suggests that targeted investment into infrastructure and training could offer an actionable strategy to improve surgical outcomes and rethink the strategies of optimal resource allocation for quality improvement in pancreas surgery worldwide.

The primary strength of this study is its global scope, reporting the largest data set of pancreas surgery outcomes, capturing variation in hospital infrastructure across 60 countries and diverse health care systems. The prospective cohort design, high center participation rate (68%), and comprehensive assessment of 21 infrastructure domains provide robust data for identifying modifiable factors. The consistency of HRI effects across sensitivity analyses, including weighted scoring, supports the robustness and generalizability of findings.

These findings align with and extend previous work showing the importance of system-level factors in surgical outcomes,^21^ consistent with work demonstrating worse surgical outcomes at lower-rated hospitals.^22^ The failure-to-rescue concept postulates that mortality differences between hospitals stem primarily from differential capacities to rescue patients from complications rather than the complication prevention itself.^11^ This study provides concrete support for this concept by identifying specific infrastructural and staffing resource elements that might enable effective rescue from severe complications. The stronger effect of HRI on mortality compared with major complications is consistent with this hypothesis, suggesting these structural resources primarily influence salvage capacity rather than initial complication occurrence.

The 6 HRI factors span multiple domains, from infrastructure, staffing, expertise, and collaboration to protocols and evidence-based practice, suggesting that optimal outcomes require coordinated excellence rather than investment in any single area. Identified factors include interventional radiology and endoscopy, which enable the management of postoperative complications such as bleeding, thereby averting reoperation and the associated mortality risks. Structured nutrition support addresses the high prevalence of malnutrition among patients undergoing pancreas surgery and may facilitate recovery and wound healing. The adoption of emerging evidence-based best practices and systematic staff training ensures the highest standards of care practices and interdisciplinary competence in managing complex care. Interestingly, adoption of evidence-based practice was significantly associated with mortality, whereas guideline adherence was not (*P*=0.066). These factors capture distinct constructs: guideline adherence reflects compliance with established protocols and standardization, while adoption of evidence-based practice reflects institutional openness to innovation and continuous improvement. In the context of failure-to-rescue, where complications are heterogeneous and management requires adaptive clinical judgment, institutional flexibility may be more predictive than rigid adherence to protocols. Equipment maintenance, while seemingly mundane, is fundamental to reliable delivery of care, particularly in time-sensitive rescue scenarios. Finally, nurse-patient ratios reflect workforce capacity for vigilant monitoring and early detection of deterioration, key to successful rescue. The HRI captures incremental protective factors, and factors with near-universal availability (98%) likely reflect fundamental importance for centers performing pancreas surgery.

The independent effects of caseload volume and infrastructure observed in the multivariable model have important implications.^23^ Volume remains a key factor in outcomes after pancreatic surgery, and our findings should not be interpreted as diminishing its importance. Indeed, high-volume centers demonstrated significantly lower mortality even after adjusting for HRI (OR: 0.73, *P*=0.014). The additional protective effect of HRI suggests that volume alone does not fully capture institutional capacity and may, in part, serve as a proxy for underlying structural factors. These findings support recent calls to look beyond volume as the sole quality metric.^24^ The identification of specific modifiable factors provides complementary targets for quality improvement that may be more achievable than centralization in some health care systems, particularly where geography or demographics preclude concentration of surgical services.

These findings reveal a critical paradox in global surgical care. While hospital resources show universal benefit across income levels, access to these resources is profoundly inequitable. HRI scores were markedly lower in centers from low- or middle-HDI countries compared to high and very-high HDI countries, reflecting systematic lower investment in surgical infrastructure in these settings. Notably, in low- and middle-HDI countries, hospital volume demonstrated a particularly pronounced protective effect, suggesting that case concentration becomes even more critical where expertise and infrastructure are scarce. These findings underscore that addressing surgical outcomes globally requires not only providing resources but also ensuring the systems and expertise to use them effectively.

The findings must be considered in the context of several limitations. Availability of hospital factors was assessed via a self-reported survey, making them subjective with potential for interpretive bias. While some HRI components are objectively verifiable (eg, on-site interventional radiology availability), others rely on subjective assessment (eg, equipment maintenance, evidence-based practice adoption). Binary response options were employed to minimize variation in interpretation across diverse settings, though this approach sacrifices granularity, and future studies should examine specific quantitative thresholds. Despite this limitation, the consistency of associations across factors and the high correlation between additive and weighted HRI scoring (*r*=0.998) support the robustness of findings. The cross-sectional assessment cannot establish causality; unmeasured confounding by factors correlated with both resources and outcomes remains possible. While we adjusted for key patient and procedural factors, residual confounding by case-mix differences across institutions cannot be excluded. Although the HRI effects were consistent across HDI strata in the interaction analysis (*P*=0.703), the limited sample from low- to middle-HDI countries (n=119, 4%) restricts definitive conclusions for these settings specifically, and future validation in low-resource contexts is warranted. Finally, the additive composite HRI does not capture potential synergistic effects between resources or the differential importance of specific factors in particular clinical contexts.

In conclusion, this study showed that specific, modifiable hospital-level factors have cumulative protective effects on mortality after pancreas surgery, independent of caseload volume and patient characteristics. The identification of 6 key resources (“interventional radiology and endoscopy on-site availability,” “structured nutritional support and counseling,” “adoption of emerging evidence-based best practice,” “systematic training of health care staff and nurses in the management of post-pancreatectomy complications,” “maintenance of equipment and facilities,” and “favorable nurse-patient ratios”) provides actionable targets for quality improvement and resource allocation beyond centralization. Given the marked global inequality in the availability of infrastructure, interventions to strengthen surgical systems in low-resource settings represent a critical priority. Future research should validate the HRI in independent cohorts, refine component definitions with quantitative thresholds (eg, nurse-to-patient ratios) and objective institutional metrics (eg, bed capacity and academic output), and evaluate whether targeted infrastructure investment translates to improved outcomes through prospective implementation studies. Understanding which resources confer the greatest benefit in specific contexts could further refine resource allocation strategies to maximize population health impact within constrained budgets.

## Supplementary Material
