# Supplementary material for: Hospital-Level Determinants of Mortality After Pancreas Surgery: A Global Infrastructure Analysis
Source: Ann Surg. 2026 Sep 9;284(5):985–92. doi: 10.1097/SLA.0000000000007196 (PMC13652114; doi:10.1097/SLA.0000000000007196)
Supplement: Supplementary file 1 [file sla-284-0985-s001.docx]

**Appendix**

**PancreasGroup.org Collaborative Authorship**

**(Name, Affiliation, Country)**

**Scientific Committee:**

Mohamed Abu Hilal, University Hospital Southampton, UK; Claudio Bassi, University Hospital of Verona, Italy; Marc Besselink, University of Amsterdam, Netherlands; Kevin Conlon, Trinity College Dublin, Ireland; Brian Davidson, University College London, UK; Marco Del Chiaro, University of Colorado Anschutz Medical Campus, USA; Christos Dervenis, Metropolitan Hospital Athens, Greece; Massimo Falconi, San Raffaele Hospital, Milano, Italy; Thilo Hackert, University of Heidelberg, Germany; Ewen Harrison, University of Edinburgh, UK; Shailesh V. Shrikhande, Tata Memorial Hospital, Mumbai, India; Ajith Siriwardena, Manchester University, UK; Martin Smith, University of the Witwatersrand, Johannesburg, South Africa.

**Management Committee**:

Aditya Borakati, Royal Free Hospital, London, UK; Deniz Balci, Ankara University Hospital, Turkey; Nikolaos Machairas, Royal Free Hospital, London, UK; Giovanni Marchegiani, University Hospital of Padua, Italy; Atsushi Oba, University of Colorado Anschutz Medical Campus, USA; Christian Oberkofler, University Hospital Zurich, Switzerland; Ioannis Passas, Metropolitan Hospital Athens, Greece; Reena Ravikumar, Royal Infirmary of Edinburgh, UK; Patricia Sánchez Velázquez, University Hospital del Mar, Barcelona, Spain; Martin de Santibañes, Italian Hospital of Buenos Aires, Argentina; Andreas Anton Schnitzbauer, University Hospital Frankfurt, Germany; Fiammetta Soggiu, Royal Free Hospital, London, UK; Domenico Tamburrino, San Raffaele Hospital, Milano, Italy; Alice Wei, Memorial Sloan Kettering Cancer, New York, USA; Marinos Zachiotis, University of Patras, Greece.

**Prospective Snapshot data contributors:**

Amor El Behi, University of Sousse, Algeria; Anisse Tidjane, Ehu-1St November 1954, Departmenet Of Medicine, University Oran 1, Algeria; Ilhem Ouahab, Ferhat Abbas University, Sétif 1, Faculty Of Medicine. Chu Saadna Abdenour., Algeria; Souad Bouaoud, Ferhat Abbas University, Sétif 1, Faculty Of Medicine. Chu Saadna Abdenour., Algeria; Mounira Rais, Ferhat Abbas University, Sétif 1, Faculty Of Medicine. Chu Saadna Abdenour., Algeria; Meriem Abdoun, Ferhat Abbas University, Sétif 1, Faculty Of Medicine. Chu Saadna Abdenour., Algeria; Amel Ouyahia, Ferhat Abbas University, Sétif 1, Faculty Of Medicine. Chu Saadna Abdenour., Algeria; Lucas Mc Cormack, Hospital Aleman Of Buenos Aires, Argentina; Martin De Santibanes, Hospital Italiano De Buenos Aires, Argentina; Pablo Barros Schelotto, Universitary Hospital Favaloro Foundation, Argentina; Shantanu Joglekar, Box Hill Hospital, Australia; Sivakumar Gananadha, Canberra Hospital, Australia; Kat Hall, Austin Health / Knox Private Hospital, Australia; Russell Hodgson, Northern Health, Australia; Alistair Rowcroft, St Vincent's Hospital Melbourne, Australia; Lynn Chong, St Vincent's Hospital Melbourne, Australia; Kalpesh Shah, John Hunter Hospital, Australia; Stanley Chen, John Hunter Hospital, Australia; David Burnett, John Hunter Hospital, Australia; Zi Qin Ng, Royal Perth Hospital, Australia; Christos Apostolou, Sydney Adventist Hospital, Australia; Peter Kornprat, Medical University Graz, Austria; Radoslava Stoyanova, Hospital Sisters Of Mercy, Austria; Aliaksei Shcherba, Minsk Medical Center For Surgery, Transplantation And Hematology, Belarus; Bert Van den Bossche, A.S.Z. Aalst - Geraardsbergen - Wetteren, Belgium; Vera Hartman, Antwerp University Hospital, Belgium; Filip Gryspeerdt, University Hospital Ghent, Belgium; Frederik Berrevoet, University Hospital Ghent, Belgium; Sébastien Strypstein, Az Delta - Az St. Jan Brugge, Belgium; Rinaldo Pinto, Hospital Santa Catarina De Blumenau, Brazil; Gustavo Coelho, Federal University Of Ceara, Brazil; Matheus Militz, Hospital De Clínicas De Porto Alegre, Brazil; Pablo Rodrigues, Ufcspa / Santa Casa Porto Alegre, Brazil; Orlando Torres, Maranhão Federal University, Brazil; Narimã Marques, AC Camargo Cancer Center, Brazil; Adriano Sampaio, Hospital Do Servidor Público Estadual De São Paulo, Brazil; Panche Krastev, UMHAT Eurohospital, Bulgaria; Mihail Slavchev, UMHAT Eurohospital, Bulgaria; Ivelin Takorov, Military Medical Academy - Sofia, Bulgaria; Nikola Vladov, Military Medical Academy - Sofia, Bulgaria; Vassil Mihaylov, Military Medical Academy - Sofia, Bulgaria; Evgeni Nikolaev, Military Medical Academy - Varna, Bulgaria; Daniel Kostov, Military Medical Academy - Varna, Bulgaria; Pablo Serrano, McMaster University, Canada; Cristian Diaz, Hospital Sotero del Río, Chile; Alejandro Brañes, Hospital Sotero del Río, Chile; Lei Cai, Chongqing General Hospital, University Of Chinese Academy Of Sciences, China; Yiming Chen, The First Affiliated Hospital Of Dali University, China; Ivan Štironja, University Hospital Merkur, Croatia; Tomislav Bubalo, University Hospital Merkur, Croatia; Martin Loveček, University Hospital Olomouc, Palacky University Olomouc;, Czech Republic (the); Pavel Zaruba, Military University Hospital Prague, Czech Republic (the); Andrej Nikov, University Hospital Kralovske Vinohrady Prague, Czech Republic (the); Almoatazbellah Attalla, Alexanderia University Hospital, Egypt; Mosaab Tayiawi, Alexanderia University Hospital, Egypt; Muneera Alboridy, Alexanderia University Hospital, Egypt; Mohamed Mourad, Alexanderia University Hospital, Egypt; Mostafa Elkeleny, Alexanderia University Hospital, Egypt; Yousef Tanas, Alexanderia University Hospital, Egypt; Hajer Ealreibi, Alexanderia University Hospital, Egypt; Mohamed Abd El Moneam, Alexanderia University Hospital, Egypt; Mohammed Hamouda, Alexanderia University Hospital, Egypt; Ahmed Nafea, Alexanderia University Hospital, Egypt; Mohamed Abdelalemm, Alexanderia University Hospital, Egypt; Ahmed Mansour, Alexanderia University Hospital, Egypt; Marina Farag, Alexanderia University Hospital, Egypt; Mohamed Salah, Al-Rajhi Liver Hospital, Egypt; Mohamed Abdelkarem, Assuit Universty Hospital, Egypt; Hatem Sayed, Ain Shams University Hospitals, Egypt; Sherein Diab, Ain Shams University Hospitals, Egypt; Mira Gobran, Ain Shams University Hospitals, Egypt; Abdelrahman Fahim, Ain Shams University Hospitals, Egypt; Ahmed K. Awad, Ain Shams University Hospitals, Egypt; Sherif Abdelmawgoud, Ain Shams University Hospitals, Egypt; Emad Alazab, Ain Shams University Hospitals, Egypt; Mostafa Nagy, Ain Shams University Hospitals, Egypt; Islam Metwally, Mansoura University, Egypt; Ahmed Shehta, Mansoura University, Egypt; Ahmed Monier, Mansoura University, Egypt; Selmy Awad, Mansoura University, Egypt; Mohammed Omar, Faculty Of Medicine, South Valley University, Egypt; Feras Alsabbagh, Zagazig University, Egypt; Joonas Kauppila, Oulu University Hospital, Finland; Minna Nortunen, Oulu University Hospital, Finland; Heikki Huhta, Oulu University Hospital, Finland; Raffaele Brustia, Henri Mondor University Hospital, France; Stéphanie Truant, Hôpital Huriez, Chu Lille, France; Guillaume Piessen, Hôpital Huriez, Chu Lille, France; Aurélien Dupré, Centre Leon Berard, France; Francois-Regis Souche, Chu Montpellier, France; Natalia Savala, Hôpital De L'Archet, Nice, France; Renato Lupinacci, Ambroise Pare Hospital, Paris-Saclay University, France; Sébastien Gaujoux, APHP, France; Nicolas Goasguen, Hopital Diaconesses/Croix Saint Simon, France; Morgan Anyla, Hôpital Pitié-Slapétrière, France; Lilian Schwarz, Rouen University Hospital, France; Fabio Giannone, Nouvel Hôpital Civil, France; Zaza Demetrashvili, Tbilisi State Medical University, Georgia; Isabel Bartella, Marienhospital Aachen, Germany; Ioannis Pozios, Charité - Universitätsmedizin Berlin, Germany; Orlin Belyaev, St. Josef Hospital, Ruhr University Bochum, Germany; Dirk Bulian, Cologne-Merheim Medical Center, Witten/Herdecke University, Germany; Christian Praetorius, University Hospital Carl Gustav Carus, Germany; Sandra Korn, University Hospital Carl Gustav Carus, Germany; Maximilian Brunner, University Hospital Erlangen, Germany; Elena Mazzella, Uniklinik Frankfurt, Germany; Martin Reichert, University Hospital Of Giessen, Germany; Jochen Gaedcke, University Medical Center Göttingen, Germany; Ulrich Ronellenfitsch, University Hospital Halle (Saale), Germany; Tim Reese, Asklepios Hospital Barmbek, Germany; Kim Wagner, Asklepios Hospital Barmbek, Germany; Pasquale Scognamiglio, University Medical Center Hamburg-Eppendorf, Germany; Faik Uzunoglu, University Medical Center Hamburg-Eppendorf, Germany; Jakob Izbicki, University Medical Center Hamburg-Eppendorf, Germany; Benjamin Struecker, Universitätsklinikum Münster (UKM), Germany; Dimitrios Kardassis, Klinikum Saarbrücken, Germany; Silvio Nadalin, University Hospital Tübingen, Germany; Georgios Makridis, St. Josefs-Hospital Wiesbaden, Germany; Nikolaos Tsoukalas, 401 General Military Hospital Of Athens, Greece; Aristotelis Kechagias, Athens Bioclinic Hospital, Greece; Argyrios Ioannidis, Athens Medical Center, Greece; Nikolaos Arkadopoulos, University General Hospital Attikon, Greece; Dimitrios Papakonstantinou, University General Hospital Attikon, Greece; Nikolaos Michalopoulos, University General Hospital Attikon, Greece; Panagiotis Kokoropoulos, University General Hospital Attikon, Greece; Pantelis Vassiliu, University General Hospital Attikon, Greece; Dimitrios Stergiou, Evangelismos General Hospital, Greece; Maria Sotiropoulou, Evangelismos General Hospital, Greece; Nefeli Tomara, Laiko General Hospital of Athens, Greece; Panagiotis Dorovinis, Laiko General Hospital of Athens, Greece; George Tzimas, Hygeia Hospital, Greece; Dimitrios Schizas, National And Kapodistrian University Of Athens, Greece; Alexandros Papalampros, National And Kapodistrian University Of Athens, Greece; Andreas Polydorou, National And Kapodistrian University Of Athens, Greece; Georgios Fragulidis, National And Kapodistrian University Of Athens, Greece; Konstantinos Toutouzas, National And Kapodistrian University Of Athens, Greece; Antonios Vezakis, National And Kapodistrian University Of Athens, Greece; Konstantinos Bramis, National And Kapodistrian University Of Athens, Greece; Dimitrios Korkolis, Saint Savvas Cancer Hospital, Greece; Evangelos Fradelos, Saint Savvas Cancer Hospital, Greece; Georgios Glantzounis, Ioannina University Hospital And School Of Medicine, University Of Ioannina, Greece; Dimitrios Magouliotis, University Hospital Of Larissa, Greece; Grigorios Christodoulidis, University Hospital Of Larissa, Greece; Francesk Mulita, University Hospital Of Patras, Greece; Elissaios Kontis, Metaxa Cancer Hospital, Greece; Vasileios Papaziogas, Aristotle University Of Thessaloniki, Greece; Dimitris Giakoustidis, Aristotle University Of Thessaloniki, Greece; Anastasios Katsourakis, General Hospital of Thessaloniki "O Agios Dimitrios", Greece; Achilleas Ntinas, Interbalkan Medical Center, Greece; Panagiotis Petras, Ippokratio General Hospital Of Thessaloniki, Greece; Attila Bursics, Uzsoki Hospital, Hungary; Andras Vereczkei, Clinical Center, Medical School University Of Pécs, Hungary; Sreenivasan Karuparthi, Sri Ramachandra Institute Of Higher Education And Research, India; Govind Purushothaman, Stanley Medical College, India; Rahul Gupta, Synergy Institute Of Medical Sciences, India; Kaushal Yadav, W Pratiksha Hospital, Gurgaon, India; Sundeep Jain, CK Birla Hospitals/ RBH Jaipur, India; Jeewan Ram Vishnoi, All India Institute Of Medical Sciences, Jodhpur, India; Sanjeev Misra, All India Institute Of Medical Sciences, Jodhpur, India; Vaibhav Varshney, All India Institute Of Medical Sciences, Jodhpur, India; Kshaunish Das, Institute Of Postgraduate Medical Education & Research, India; Somnath Chattopadhyay, Kokilaben Dhirubhai Ambani Hospital, India; Jayapala Reddy Velagala, Kokilaben Dhirubhai Ambani Hospital, India; Varun Bansal, Seth G.S Medical College And K.E.M Hospital, India; Induchoodan S, Government Medical College, Thiruvananthapuram, India; Tarun Kumar, Institute Of Medical Sciences, Banaras Hindu University, Varanasi, India; Erik Prabowo, Diponegoro University / Kariadi General Hospital, Indonesia; Hashim Talib Hashim, University Of Baghdad, College Of Medicine, Iraq; Maytham Al-Juaifari, Al-Sader Teaching Hospital, Najaf, Iraq; Eran Sadot, Rabin Medical Center, Israel; Marco Vivarelli, Marche Polytechnic University, Italy; Valeria Andriola, Policlinico Di Bari, Italy; Michele Ciola, Regional Hospital Bolzano/Bozen, Italy; Mario Virgilio Papa, AORN Caserta, Italy; Adelmo Antonucci, Sant'Anna Hospital, Italy; Diego Sasia, Santa Croce And Carle Hospital, Italy; Valentina Testa, Santa Croce And Carle Hospital, Italy; Lapo Bencini, Azienda Ospedaliero-Universitaria Careggi, Italy; Tommaso Nelli, Ospedale San Giovanni Di Dio, Italy; Fabrizio D'Acapito, Ausl Romagna, Morgagni-Pierantoni Hospital, Italy; Carlo Alberto Pacilio, Ausl Romagna, Morgagni-Pierantoni Hospital, Italy; Giacomo Carganico, Ospedale San Martino, Italy; Raffaele De Rosa, University Of Genoa, Italy; Stefano D'Ugo, Division Of HPB Surgery - "Vito Fazzi" Hospital, Italy; Edoardo Saladino, Azienda Ospedaliera Papardo, Italy; Alfonso Recordare, Dell'Angelo Hospital, Italy; Michele Mazzola, Asst Grande Ospedale Metropolitano Niguarda, Italy; Vincenzo Mazzaferro, Fondazione IRCCS Istituto Nazionale Tumori, Italy; Alessandro Zerbi, Humanitas Research Hospital, Italy; Domenico Tamburrino, IRCCS San Raffaele Hospital, Vita-Salute University, Italy; Caterina Baldi, San Carlo Borromeo Hospital, University Of Milano, Italy; Marco Stella, San Carlo Borromeo Hospital, University Of Milano, Italy; Fabrizio Di Benedetto, University Of Modena And Reggio Emilia, Italy; Fabio Uggeri, IRCCS Foundation San Gerardo dei Tintori, Italy; Alessandro Iacomino, Ospedale Antonio Cardarelli, Italy; Gianluca Rompianesi, Federico II University Hospital, Italy; Andrea Belli, Istituto Nazionale Tumori - IRCCS - Fondazione G. Pascale, Italy; Lucia Moletta, University Of Padova, Italy; Francesca Tolin, Veneto Institute Of Oncology IOV – IRCCS, Italy; Mario Giuffrida, Parma University Hospital, Italy; Lorenzo Cobianchi, University Of Pavia, Italy; Paolo Regi, Pederzoli Hospital, Italy; Luca Morelli, University Of Pisa, Italy; Emanuele Federico Kauffmann, University Of Pisa, Italy; Enrico Pinotti, Policlinico San Pietro, Italy; Felice Giuliante, Fondazione Policlinico Universitario Agostino Gemelli IRCCS, Italy; Alessandro Coppola, Fondazione Policlinico Universitario Campus Bio-Medico, Italy; Tommaso Maria Manzia, Tor Vergata University of Rome, Italy; Alberto Porcu, Azienda Ospedaliero Universitaria di Sassari, Italy; Claudio Feo, Azienda Ospedaliero Universitaria di Sassari, Italy; Teresa Perra, Azienda Ospedaliero Universitaria di Sassari, Italy; Francesco Ciarleglio, Santa Chiara Hospital - APSS, Italy; Simone Novello, IV Chirurgia, Ospedale Di Treviso; Università Di Padova, Italy; Alessandro Ferrero, Ospedale Mauriziano, Italy; Sergio Intini, Università Degli Studi Di Udine - ASUFC Ospedale Santa Maria Della Misericordia, Italy; Giorgio Querini, Ospedale "Castelli", Verbania - ASL VCO, Italy; Andrea Ruzzenente, The Pancreas Institute, University of Verona, Italy; Tommaso Campagnaro, The Pancreas Institute, University of Verona, Italy; Simone Conci, The Pancreas Institute, University of Verona, Italy; Sara Napetti, San Bortolo Hospital, Italy; Alice Frontali, ASST Brianza - Vimercate Hospital, Italy; Daisuke Hashimoto, Kansai Medical University, Japan; Ippei Matsumoto, Kindai University, Japan; Hiromitsu Maehira, Shiga University Of Medical Science, Japan; Minoru Tanabe, Tokyo Medical And Dental University, Japan; Subhi Alissawi, Al-Basheer Hospital, Jordan; Aiman Obed, Jordan University Hospital, Jordan; Hebah Rababa, Jordan University Hospital, Jordan; Khayry Al-Shami, Jordan University Hospital, Jordan; Mohamed Alsabah, King Abdullah University Hospital, Jordan; Saeed Shumrakh, King Abdullah University Hospital, Jordan; Abdulaziz Al-Samawi, King Abdullah University Hospital, Jordan; Almu'Atasim Khamees, Yarmouk University - Irbid, Jordan; Ildar Fakhradiyev, KAZNMU, Kazakhstan; Ho-Seong Han, Seoul National University Bundang Hospital, Korea (the Republic of); Kristaps Atstupens, Riga East Clinical University Hospital, Latvia; Walid Faraj, American University Of Beirut, Lebanon; Mohamad Khalife, American University Of Beirut, Lebanon; Fatoom Alowjali, Banghazi Aljala Hospital, Libya; Abdulhadi Alshatshat, National Cancer Institute, Libya; Aihab Benamwor, National Cancer Institute, Libya; Eman Younes, National Cancer Institute Of Sabratah, Libya; Dania Burgan, National Cancer Institute, Sabratha - Libya, Libya; Marwa Morgom, Alestiklal Hospital At Libya, Libya; Muhannud Binnawara, Tripoli Central Hospital, Libya; Entisar Alshareea, Tripoli Central Hospital, Libya; Sultan Ahmeed Ahmeed, Tripoli Central Hospital, Libya; Eman Othman, Tripoli Central Hospital, Libya; Ahmed Gerwash, Tripoli Central Hospital, Libya; Osama Salem, Tripoli Medical Center, Libya; Wegdan Khalil, Tripoli Medical Center, Libya; Eman Abdulwahed, Tripoli Medical Center, Libya; Mohamed Alharari, Tripoli Medical Center, Libya; Tomas Vanagas, Lithuanian University Of Health Sciences, Lithuania; Vitalijus Eismontas, Klaipeda University Hospital, Lithuania; Jonas Jurgaitis, Klaipeda University Hospital, Lithuania; Algirdas Slepavicius, Klaipeda University Hospital, Lithuania; Vytenis Mikutaitis, Klaipeda University Hospital, Lithuania; Mindaugas Kvietkauskas, Vilnius University Hospital Santaros Klinikos, Lithuania; Edoardo Rosso, Centre Hospitalier Du Luxembourg, Luxembourg; Andee Dzulkarnaen Zakaria, School Of Medical Sciences & Hospital, Universiti Sains Malaysia, Malaysia; Ian Chik, Universiti Kebangsaan Malaysia Medical Centre, Malaysia; Jin Bong, Sunway Medical Center, Malaysia; Carlos Florez Zorrilla, Centro Medico Nacional 20 De Noviembre, Mexico; Sarnai Erdene, Mongolian National University Of Medical Sciences, Mongolia; Moniba Korch, University Hospital Mohammed VI, Morocco; Iltimass Gouazar, Faculté De Médecine Et De Pharmacie De Marrakech University Hospital Center Mohamed, Morocco; Badr Serji, Oncology Hospital Hasssan II. Faculty Of Medicine And Pharmacy Mohammed Ist University, Morocco; Aziz Zentar, Military Universitaire Hospital Med V Rabat Morocco, Morocco; Amine Benkabbou, National Institute Of Oncology, Mohammed V University In Rabat, Morocco; Reda Elhassouni, National Institute Of Oncology, Mohammed V University In Rabat, Morocco; Sabrillah Echiguer, National Institute Of Oncology, Mohammed V University In Rabat, Morocco; Mohammed Anass Majbar, National Institute Of Oncology, Mohammed V University In Rabat, Morocco; Abdulrashid Pueya Nashidengo, Windhoek Central Academic Hospital/University Of Namibia School Of Medicine, Namibia; Paleswan Joshi Lakhey, Tribhuvan University Teaching Hospital, Institute Of Medicine, Nepal; John Windsor, Auckland City Hospital, New Zealand; Michael Jen Jie Chu, Auckland City Hospital, New Zealand; Peter Johnston, Auckland City Hospital, New Zealand; Vanshay Bindra, Waitemata District Health Board, New Zealand; Andrea Cross, Christchurch Hospital, New Zealand; Ashok Gunawardene, Waikato Hospital, New Zealand; Fraser Welsh, Waikato Hospital, New Zealand; Olusegun Alatise, Obafemi Awolowo University, Nigeria; Jibran Abbasy, Aga Khan University Hospital, Pakistan; Tayyab Siddiqui, Aga Khan University Hospital, Pakistan; Mohammad Asghar, King Edward Medical University Mayo Hospital Lahore, Pakistan; Mustafa Abu Jayyab, Alshifaa Hospital, Palestine, State of; Qusai Zreqat, Al-Ahli Hospital-Hebron-Palestine, Palestine, State of; Rawand Titi, Alia Governmental Hospital -Hebron, Palestine, State of; Fatima Manasrah, Rafedia Government Hospital, Palestine, State of; Ahlam Hammoudeh, Palestine Medical Complex, Palestine, State of; Guillermo Coayla, Hospital Nacional Edgardo Rebagliati Martins, Peru; Catherine Teh, Makati Medical Center, Philippines (the); Cenon Alfonso, Ateneo School Of Medicine And Public Health, Dean, Philippines (the); Marta Flisińska, Jurasz University Hospital, Poland; Justyna Rymarowicz, University Hospital Krakow, Poland; Marek Sierzega, Jagiellonian University Medical College, Poland; Wojciech Ciesielski, Medical University Of Lodz, Poland; Oliwia Grząsiak, Medical University Of Lodz, Poland; Patrycja Szewczyk, Medical University Of Lodz, Poland; Krzysztof Szwedziak, Medical University Of Lodz, Poland; Wojciech Korcz, Medical University Of Warsaw, Poland; Oskar Kornasiewicz, Oskar Kornasiewicz, Poland; Emanuel Vigia, Hospital De Curry Cabral - Centro Hospitalar Universitário De Lisboa Central, Portugal; Raluca Bievel Radulescu, Emergency Hospital Prof Dr. “Agrippa Ionescu”, Romania; Traian Dumitrascu, Fundeni Clinical Institute, Romania; Mara Mardare, Monza Hospital Bucharest, Romania; Octav Ginghina, Saint John Emergency Hospital, Romania, Romania; Iulian Brezean, Spitalul Clinic Dr. I. Cantacuzino, Romania; Sorin Petrea, Spitalul Clinic Dr. I. Cantacuzino, Romania; Adrian Bartos, Iuliu Hatieganu University Of Medicine And Pharmacy, Romania; Raluca Bodea, Regional Institute Of Gastroenterology And Hepatology, Romania; Sergiu Matei, University Hospital C.F.R. Cluj-Napoca, Romania, Romania; Ana-Maria Musina, Regional Institute Of Oncology, Romania; Natalia Velenciuc, Regional Institute Of Oncology, Romania; Alexander Belyaev, Archangel Regional Clinical Hospital, Russian Federation; Denis Mizgirev, First Clinical City Hospital Named After E. E. Volosevich, Russian Federation; Andrey Litvin, Immanuel Kant Baltic Federal University, Regional Clinical Hospital, Russian Federation; Ivan Semenenko, I.M. Sechenov First Moscow State Medical University (Sechenovskiy University), Russian Federation; Arkady Bedzhanyan, Petrovsky National Research Center Of Surgery, Russian Federation; Nikolay Bagmet, Petrovsky National Research Center Of Surgery, Russian Federation; Ayrat Kaldarov, Vishnevsky Center Of Surgery, Russian Federation; Denis Kuchin, Privolzhsky District Medical Center, Russian Federation; Evgeniy Drozdov, Siberian State Medical University, Russian Federation; Mahir Gachabayov, Vladimir City Emergency Hospital, Russian Federation; Mohammed Alharthi, King Abdulaziz University Hospital, Saudi Arabia; Aleksandar Bogdanovic, University Clinical Center of Serbia, Serbia; Daniel Galun, University Clinical Center of Serbia, Serbia; Stefan Kmezic, University Clinical Center of Serbia, Serbia; Dragana Arbutina, KBC Zvezdara Klinika Za Hirurgiju "Nikola Spasić", Serbia; Mihailo Bezmarevic, Military Medical Academy, Serbia; Jovan Juloski, Zvezdara University Medical Center, Serbia; Mladjan Protic, Oncology Institute Of Vojvodina, Serbia; Brian Goh, Singapore General Hospital, Singapore; Blaž Trotovšek, University Medical Centre Ljubljana, Slovenia; Emil Loots, Entabeni Hospital, South Africa; Jones Omoshoro-Jones, Chris Hani Baragwanath Academic Hospital/University Of The Witwatersrand, South Africa; Manuel Marcello, Hospital Universitario Fundación Alcorcón, Spain; Jose Ramia, Hospital General Universitario de Alicante Dr. Balmis, Spain; Maria Del Mar Rico-Morales, Hospital Universitario Torrecárdenas, Spain; Fabio Ausania, Hospital Clinic, IDIBAPS, University of Barcelona, Spain; Ana Belen Martin Arnau, Hospital de la Santa Creu i Sant Pau, Spain; Benedetto Ielpo, Hospital Del Mar, Spain; Esther Pilar Santos, Hospital General Universitario De Ciudad Real, Spain; Patricia Ruiz, Cruces University Hospital, Spain; Laia Falgueras, Hospital Universitari De Girona Dr. Josep Trueta, Spain; Farah Al Shwely, Hospital University Guadalajara, Spain; Juli Busquets, Bellvitge University Hospital, Spain; Iago Justo, "12 De Octubre" University Hospital, Spain; Jana Dziakova, Hospital Clinico San Carlos, Spain; Marcello Di Martino, Hospital Universitario La Princesa, Spain; Miguel Ángel Suárez-Muñoz, University Hospital Virgen de la Victoria, Spain; Lorena Solar García, Hospital Universitarios Central de Asturias, Spain; Juan Jose Segura-Sampedro, Hospital Son Espases, Spain; Fernando Rotellar, Clinica Universidad De Navarra, Spain; Luis Muñoz-Bellvis, Hospital Universitario De Salamanca. Universidad De Salamanca, Spain; Valle Vera, Hospital Quiron Salud Infanta Luisa, Spain; María García Domingo, Hospital Universitari Mutua Terrassa, Spain; Carlos Domingo-Del Pozo, Hospital Universitario Dr. Peset, Spain; Cristina Ballester Ibáñez, Hospital Universitario La Fe, Spain; Dimitri Dorcaratto, Hospital Clínico Universitario de Valencia, Spain; Mario Rodriguez-Lopez, Hospital Clínico Universitario de Valladolid, Spain; Alejandro Serrablo, Miguel Servet University Hospital, Spain; Hytham Hamid, Ibn Sina Hospital, Sudan; Abdelfatah Abdelmageed, Omdurman Islamic University, Sudan; Per Sandström, University Hospital Of Linköping, Sweden; Linda Lundgren, University Hospital Of Linköping, Sweden; Bobby Tingstedt, Lund University, Sweden; Caroline Williamsson, Lund University, Sweden; Bodil Andersson, Lund University, Sweden; Ernesto Sparrelid, Karolinska Institutet, Sweden; Christoph Kuemmerli, Clarunis, St. Clara Hospital And University Hospital., Switzerland; Beat Moeckli, Hôpitaux Universitaire de Genève, Switzerland; Alessandra Cristaudi, Ospedale Regionale Di Lugano, Switzerland; Fariba Abbassi, Kantonsspital St.Gallen, Switzerland; Jan Schmidt, Hirslanden Clinics Zurich, Switzerland; Stefan Gutknecht, Triemli Hospital Zurich, Switzerland; Jan Philipp Jonas, University Hospital Zurich, Switzerland; Mais Alhashemi, University Of Aleppo, Syrian Arab Republic; Zain Douba, University Of Aleppo, Syrian Arab Republic; Ahmad Kayali, University Of Aleppo, Syrian Arab Republic; Oula Azizeh, University Of Aleppo, Syrian Arab Republic; Omar Al-Abed, University Of Aleppo, Syrian Arab Republic; Aya Abdulmonem, Ain Shams University Hospitals, Syrian Arab Republic; Ahmad Alhouri, Al Assad University Hospital, Syrian Arab Republic; Hasan Al Houri, Al Assad University Hospital, Syrian Arab Republic; Alnour Suliman, Alasad Hospital, Syrian Arab Republic; Bashar Haj Hassan, Tishreen University Hospital, Syrian Arab Republic; Alaa Hamdan, Tishreen University Hospital, Syrian Arab Republic; Ming-Chin Yu, New Taipei Municipal Tucheng Hospital, Chang Gung Medical Foundation, Taiwan (Province China); Hanen Bouaziz, Salah Azaiez Institut, Tunisia; Ali Kchaou, Habib Bourguiba University Hospital, Tunisia; Ammar Houssem, Sahloul Hospital, Tunisia; Mesut Tez, Ankara Numune Hospital, Turkey; Mustafa Kerem, Gazi University, Turkey; Hüseyin Bayhan, Gazi University, Turkey; Ulaş Aday, Dicle University School Of Medicine, Turkey; Alpen Gumusoglu, Bakırkoy Dr. Sadi Konuk Research And Training Hospital, Turkey; Husnu Aydin, Bakırkoy Dr. Sadi Konuk Research And Training Hospital, Turkey; Fatema Hanefa, Istanbul University Faculty Of Medicine (Çapa), Turkey; Ali Emre Atici, Marmara University, Turkey; Hanife Ulgur, Ümraniye Training and Research Hospital, Turkey; Ismail Sert, Egepol Surgery Hospital, Turkey; Semra Demirli Atici, University Of Health Sciences Tepecik Training And Research Hospital, Turkey; Elif Colak, Samsun Training And Research Hospital, Turkey; Denys Skoryi, Kharkiv Regional Center Of Oncology, Ukraine; Kostiantyn Kopchak, National Cancer Institute, Ukraine; Oleksandr Kvasivka, National Cancer Institute, Ukraine; Valeriia Sumarokova, National Cancer Institute, Ukraine; James Skipworth, University Hospitals Bristol NHS Trust, United Kingdom; William Lim, University Of Glasgow, United Kingdom; Ahmed Mohamed, Royal Free London NHS Foundation Trust, United Kingdom; Pascale Tinguely, Royal Free London NHS Foundation Trust, United Kingdom; Hemant Kocher, The London Clinic, United Kingdom; Amjad Khalil, Wellington Hospital, HCA, United Kingdom; Nicola de' Liguori Carino, Manchester Royal Infirmary, University Of Manchester, United Kingdom; Fahed Gareb, Queen Elizabeth Queen Mother Hospital, United Kingdom; Pandanaboyana Sanjay, Freeman Hospital, United Kingdom; Krishnakumure Patel, Nottingham University Hospitals, United Kingdom; Carlo Ceresa, Oxford University Hospitals NHS Foundation Trust, United Kingdom; Thomas Russell, University Hospitals Plymouth NHS Trust, United Kingdom; Matt Mortimer, Morriston Hospital, United Kingdom; Megan Sulciner, Brigham And Women's Hospital, United States of America; Thomas Clancy, Brigham And Women's Hospital, United States of America; Martina Nebbia, Massachusetts General Hospital, United States of America; Rachel Thompson, Baylor University Medical Center, United States of America; Dimitrios Moris, Duke University Medical Center, United States of America; Jennifer Gnerlich, Inova Schar Cancer Institute, United States of America; Richard Spencer-Cole, University Of Arkansas For Medical Sciences, United States of America; Derek Krinock, University Of Arkansas For Medical Sciences, United States of America; Tamara Osborn, University Of Arkansas For Medical Sciences, United States of America; Hailey Hardgrave, University Of Arkansas For Medical Sciences, United States of America; Joe Nigh, University Of Arkansas For Medical Sciences, United States of America; Beth Schrope, Columbia University, United States of America; Ryan Lamm, Thomas Jefferson University Hospital, United States of America; Harish Lavu, Thomas Jefferson University Hospital, United States of America; Wilbur Bowne, Thomas Jefferson University Hospital, United States of America; Jonathan Sham, University Of Washington, United States of America; Paulo Martins, University Of Massachusetts, United States of America; Aisha Albar, Sana'a University, Al-Thawra Modern General Hospital, Yemen; Fatima Al-Eryani, Sana'a University, Al-Thawra Modern General Hospital, Yemen; Hamza Al-Naggar, Sana'a University, Al-Thawra Modern General Hospital, Yemen; Rafat Al-Saban, Sana'a University, Al-Thawra Modern General Hospital, Yemen.

**Survey respondents:**

Chafik Bouzid, Anti Cancer Center, Algeria; Juba Mansouri, Ehu-1St November 1954, Departmenet Of Medicine, University Oran 1, Algeria; Meharzi Sif el islam, Ehu-1St November 1954, Departmenet Of Medicine, University Oran 1, Algeria; Anisse Tidjane, Ehu-1St November 1954, Departmenet Of Medicine, University Oran 1, Algeria; Ilhem Ouahab, Ferhat Abbas University, Sétif 1, Faculty Of Medicine. Chu Saadna Abdenour., Algeria; Ezequiel Anibal Silberman, Clínica Pasteur, Argentina; Lucas McCormack, Hospital Aleman, Argentina; Paul Eduardo Lada, Hospital Nacional de Clinicas, Argentina; Ignacio Guillermo Merlo, Instituto Alexander Fleming, Argentina; Martin Cersosimo, Instituto Medico Adrogue, Argentina; Leonardo Agustin Montes, Universitary Hospital Favaloro Foundation, Argentina; Mehrdad Nikfarjam, Austin Health, University of Melbourne, Australia; Neil Merrett, Bankstown-Lidcombe Hospital, Australia; Sivakumar Gananadha, Canberra Health Services, Australia; S. George Barreto, Flinders Medical Centre, Australia; Ramesh Damodaran Prabha, Gold Coast University Hospital, Australia; David Burnett, John Hunter Hospital, Australia; Mithra Sritharan, Monash Health, Australia; Sameer Mihrshahi, MQ Health, Macquarie University Hospital, Australia; Russell Hodgson, Northern Health, Australia; Jonathan Sivakumar, Peter MacCallum Cancer Centre, Australia; Nicholas O’Rourke, Royal Brisbane and Women's Hospital, Australia; Kai Brown, Royal North Shore Hospital, Australia; Anubhav Mittal, Royal North Shore Hospital, Australia; Jaswinder Singh Samra, Royal North Shore Hospital, Australia; Suresh Navadgi, Royal Perth Hospital, Australia; Mikael Johansson, Sir Charles Gairdner Hospital, Australia; Oliver Maximilian Fisher, St George Hospital, Australia; Christos Apostolou, Sydney Adventist Hospital, Australia; Christopher Bahdah Nahm, The University of Sydney, Australia; Guy John Maddern, University of Adelaide, Australia; Wayne J Hawthorne, University of Sydney, Westmead Hospital, Australia; Tony Pang, Westmead Hospital, Australia; Thomas Gruenberger, Clinic Favoriten, Austria; Peter Kornprat, Division of General.-Viszeral and Transplantation Surger, Austria; Radoslava Petkova Stoyanova, Hospital Sisters of Mercy (Barmherzige Schwestern Krankenhaus Wien), Austria; Reinhold Johann Klug, LK Horn, Austria; Stefan Stättner, OÖG, Austria; Dmitry Mikhailovich Adamovich, Gomel State Medical University, Belarus; Valentin Bereshchenko, Gomel State Medical University, Belarus; Andrey Litvin, Gomel State Medical University, Belarus; Aliaksei Shcherba, Minsk Medical Center for Surgery transplantation and Hematology, Belarus; Bert Van den Bossche, ASZ Aalst, Belgium; Sébastien Strypstein, AZ Delta, Belgium; Franky Paul Vansteenkiste, AZ Groeninge Hospital, Belgium; Edward Willems, AZ Groeninge Hospital, Belgium; Claude Bertrand, CHU UCL Namur - Godinne, Belgium; Catherine Hubert, Cliniques universitaires saint Luc, Belgium; Vera Hartman, University Hospital Antwerp, Belgium; Bart Hendrikx, University Hospital Antwerp, Belgium; Dirk Karel Ysebaert, University Hospital Antwerp, Belgium; Frederik Berrevoet, University Hospital Ghent, Belgium; Filip Gryspeerdt, University Hospital Ghent, Belgium; Thomas Apers, ZAS, Belgium; Mauricio Pasten, Bolivian Japanese institute of gastroenterology, Bolivia; João Emílio Lemos Pinheiro Filho, ABC Medical School, Brazil; Felipe José Fernández Coimbra, AC Camargo Cancer Center, Brazil; Paulo Everton Garcia Costa, Federal University Of Ceara, Brazil; Alessandro Bersch Osvaldt, Hospital de Clínicas de Porto Alegre, Universidade Federal do Rio Grande do Sul, Brazil; Adriano Pereira Sampaio, Hospital Do Servidor Público Estadual De São Paulo, Brazil; Gustavo Mendonça Ataíde Gomes, Hospital Geral Dagoberto Omena, Brazil; Omero Pereira Costa Filho, Hospital Militar de Área de Porto Alegre, Hospital de Clínicas de Porto Alegre, Universidade Luterana do Brasil, Brazil; Rinaldo Danesi Pinto, Hospital Santa Catarina de Blumenau, Brazil; Marciano Anghinoni, Hospital São Marcelino Champagnat, Brazil; Marcel Autran Machado, Nove de julho hospital, Brazil; Pablo Duarte Rodrigues, Ufcspa / Santa Casa Porto Alegre, Brazil; Anis Ahmad, Ripash, Brunei; Radoslav Kostadinov, Military Medical Academy - Sofia, Bulgaria; Tsonka Veselinova Lukanova, Military Medical Academy - Sofia, Bulgaria; Vassil Ivanov Mihaylov, Military Medical Academy - Sofia, Bulgaria; Tsvetan Trichkov, Military Medical Academy - Sofia, Bulgaria; Daniel Vasilev Kostov, Military Medical Academy - Varna, Bulgaria; Evgeni Mladenov Nikolaev, Military Medical Academy - Varna, Bulgaria; Vasil Danielov Kostov, Sofia Med, Bulgaria; Plamen Milchev Chernopolsky, St.. Marina Hospital, Bulgaria; Alexander Julianov, Trakia Hospital, Bulgaria; Azize Saroglu, Trakia Hospital, Bulgaria; Panche Krastev, UMHAT Eurohospital, Bulgaria; Mihail Slavchev, UMHAT Eurohospital, Bulgaria; Elena Rosenova Arabadzhieva, University Hospital Alexandrovska, Bulgaria; Anna Vladimirova Taseva, University Hospital Alexandrovska, Bulgaria; Raffaello Roesel, Centre Hospitalier de l'Université de Montréal (CHUM), Canada; Franck Vandenbroucke-Menu, Centre Hospitalier de l'Université de Montréal (CHUM), Canada; Prosanto Chaudhury, McGill University Health Center, Canada; Pablo E Serrano, McMaster University, Canada; Sean Cleary, University Health Network (UHN) - Toronto, Canada; Julie Hallet, University Health Network (UHN) - Toronto, Canada; Carol-Anne Moulton, University Health Network (UHN) - Toronto, Canada; Chaya Shwaartz, University Health Network (UHN) - Toronto, Canada; Elijah Dixon, University of Calgary, Canada; Juan Glinka, Western University, Canada; Erwin Buckel, Hospital Sotero del Río, Chile; Cristian Diaz, Hospital Sotero del Río, Chile; Nicolas Andres Devaud, Instituto Oncologico Fundacion Arturo Lopez Perez (FALP), Chile; Jose Costa-Maia, Centro Hospitalar Conde de S Januario, China; Hui Liang, Second Affiliated Hospital Of Nanchang University, China; Yiming Chen, The First Affiliated Hospital of Dali University, China; Carlos Alberto Millan, Hospital San José, Colombia; Marco José Quintero, Imat Auna, Colombia; Tomislav Bubalo, KBC Zagreb, Croatia; Mario Kopljar, University Hospital Center Sisters of Charity, Croatia; Ivan Romic, University Hospital Centre Zagreb, Croatia; Mislav Rakić, University Hospital Dubrava, Croatia; Ante Šabić, University Hospital Dubrava, Croatia; Danko Mikulic, University Hospital Merkur, Croatia; Ivan Štironja, University Hospital Merkur, Croatia; Alberto Suárez Cuevas, Instituto Nacional de Oncologia y Radiobiología, Cuba; Villamil Martínez Ramón, William Soler Pediatric Hospital, Cuba; Athanasios Petrou, European University of Cyprus - School of Medicine, Cyprus; Panayiotis Papatheodorou, Nicosia General Hospital, Cyprus; Nikolaos Gouvas, University of Cyprus, Cyprus; Jiri Fronek, Institute for Clinical and Experimental Medicine, Czechia; Martin Straka, Nemocnice AGEL Novy Jicin, Czechia; Stefan Kobbelgaard Burgdorf, Center of Cancer and Transplantation, Denmark; Michael Bau Mortensen, Odense University Hospital, Denmark; Carsten Palnaes Hansen, Rigshospitalet Copenhagen University Hospital, Denmark; Luit Penninga, Rigshospitalet Copenhagen University Hospital, Denmark; Christoph Tschuor, Rigshospitalet Copenhagen University Hospital, Denmark; Alex E. Vasconez Garcia, Clínica Pasteur, Ecuador; Sherif Emad Abdelmawgoud, Ain Shams University Hospitals, Egypt; Ahmed K Awad, Ain Shams University Hospitals, Egypt; Mohamed Elnagar, Ain Shams University Hospitals, Egypt; Mira Gobran, Ain Shams University Hospitals, Egypt; Hatem Sayed Saber, Ain Shams University Hospitals, Egypt; A Abo-elmagd, Alexandria University Hospital, Egypt; Khaled Mohamed Abbasia, Alexandria University Hospital, Egypt; Yomna E. Dean, Alexandria University Hospital, Egypt; Mostafa Refaie Elkeleny, Alexandria University Hospital, Egypt; Ahmed Mohamed Elshafie, Alexandria University Hospital, Egypt; Mohammed Hamouda, Alexandria University Hospital, Egypt; Yasmine Mohamed Hasaballah, Alexandria University Hospital, Egypt; Mariam Maher Kassem, Alexandria University Hospital, Egypt; Mohamed Alsayed Mabrouk, Alexandria University Hospital, Egypt; Ahmed Mohammed Farid Mahmoud Hamdy Mansour, Alexandria University Hospital, Egypt; Mohamed I. Mohamed, Alexandria University Hospital, Egypt; Mai Adly Mohamed, Alexandria University Hospital, Egypt; Yousef Tanas, Alexandria University Hospital, Egypt; Mohamed Zein, Alexandria University Hospital, Egypt; Ahmed Elewa Abdelwahab, Assistant lecturer, Egypt; Ahmed M. Abbas, Assiut University Hospital, Egypt; Tameem Ibraheem, Assiut University Hospital, Egypt; Reem Sayad, Assiut University Hospital, Egypt; Mohamed Allam, Benha University hospitals, Egypt; Mahmoud Elfiky, Cairo University, Egypt; Khaled Amer, International Medical Center (IMC) Cairo, Egypt, Egypt; Amr Ahmed Sanad, Mansoura University, Egypt; Selmy Sabry Awad, Mansoura University, Egypt; Ahmed Shehta, Mansoura University (Gastrointestinal Surgery Center), Egypt; Islam H Metwally, Mansoura University (Oncology center), Egypt; Amro Mohamed Shawkat, Minia University, Egypt; Mohammed Esmat Bakhiet, New valley university, Egypt; Emad Ali Ahmed, Sohag university, Egypt; Khalid Mohammed Rezk, South Egypt cancer Institute, Egypt; Zeki Abdurahman Abubeker, Addis Ababa university, medical faculty, Ethiopia; Tuomo Kalevi Rantanen, Kuopio University Hospital, Finland; Minna Nortunen, Oulu University Hospital, Finland; Jean-Marc Regimbeau, Amiens Universitary Hospital, France; Sébastien Gaujoux, APHP, France; Safi Dokmak, Beaujon hospital, France; Aurélien Dupré, Centre Léon Bérard, France; Alexandre Doussot, CHU Besançon, France; Frédéric Borie, CHU de Nîmes, France; Antonio Castaldi, CHU de Nîmes, France; Rami Rhaiem, CHU de Reims, France; Regis Souche, Chu Montpellier, France; Fabrice Muscari, CHU Rangueil, France; Johanna Zemour, CHU Sud de la Réunion, France; Mustapha Adham, Edouard Herriot Hospital, France; Thomas Zacharias, Hôpital Emile Muller, GHRMSA, France; Truant Stephanie, Hôpital Huriez, Chu Lille, France; Charles de Ponthaud, Hôpital Pitié-Slapétrière, France; Tullio Piardi, HPB unit, France; Olivia Sgarbura, Institut du Cancer Montpellier, France; Jonathan Garnier, Institut Paoli Calmettes, France; Fabio Giannone, Nouvel Hôpital Civil (University Hospital of Strasbourg), France; Jerome Danion, University hospital Center, France; Johan Gagniere, University Hospital of Clermont-Ferrand, France; Raffaele De Rosa, University hospital Rennes, France; Laurent Sulpice, University hospital Rennes, France; Andrew Alexander Gumbs, American Hospital Tbilisi, Georgia; Zaza Demetrashvili, Tbilisi State Medical University, Georgia; Kim Caroline Wagner, Asklepios Klinik Barmbek, Germany; Ioannis Pozios, Charité - Universitätsmedizin Berlin, Germany; Dimitrios Kardassis, Klinikum Saarbrücken, Germany; Gregor Alexander Stavrou, Klinikum Saarbrücken, Germany; Lazaros Lazarou, Klinikum Stuttgart, Germany; Pawel Ryszard Mroczkowski, Knappschafts University Hospital Bochum Langendreer, Germany; Daniel Seehofer, Leipzig University Medical Center, Germany; Jorg Kleeff, Martin-Luther-University Halle-Wittenberg, Germany; Carsten Kamphues, Park-Klinik Weißensee, Germany; Orlin Belyaev, St. Josef Hospital, Ruhr University Bochum, Germany; Georgios Makridis, St. Josefs-Hospital Wiesbaden, Germany; Matthias Anthuber, University Hospital Augsburg, Germany; Marius Distler, University Hospital Carl Gustav Carus TU Dresden, Germany; Olga Radulova-Mauersberger, University Hospital Carl Gustav Carus TU Dresden, Germany; Maximilian Brunner, University Hospital Erlangen, Germany; Robert Grutzmann, University Hospital Erlangen, Germany; Christian Krautz, University Hospital Erlangen, Germany; Artur Rebelo, University Hospital Halle (Saale), Germany; Ulrich Ronelleniftsch, University Hospital Halle (Saale), Germany; Andreas Hecker, University Hospital of Giessen, Germany; Martin Reichert, University Hospital of Giessen, Germany; Silvio Nadalin, University Hospital Tübingen, Germany; Emrullah Birgin, University Hospital Ulm, Germany; Joerg C Kalff, University Medical Center Bonn, Germany; Jakob Robert Izbicki, University Medical Center Hamburg-Eppendorf, Germany; Felix Nickel, University Medical Center Hamburg-Eppendorf, Germany; Faik G. Uzunoglu, University Medical Center Hamburg-Eppendorf, Germany; Thaer S. A. Abdalla, University Medical Center Schleswig-Holstein, Campus Lübeck, Germany; Dirk Rolf Bulian, Witten/Herdecke University, Germany; Dimitrios Politis, Aretaieion University Hospital, Greece; Elissavet Anestiadou, Aristotle University Of Thessaloniki, Greece; Orestis Ioannidis, Aristotle University Of Thessaloniki, Greece; Apostolos Georgios Kamparoudis, Aristotle University Of Thessaloniki, Greece; Dimitrios Raptis, Aristotle University Of Thessaloniki, Greece; Georgios Tsoulfas, Aristotle University Of Thessaloniki, Greece; Argyrios Ioannidis, Athens Medical Center, Greece; Aristotelis Kechagias, Athens Metropolitan General Hospital, Greece; Dimitrios Balalis, Athens Naval Hospital, Greece; Vasileios Kalles, Athens Naval Hospital, Greece; Ioannis Kyriazanos, Athens Naval Hospital, Greece; Dimitrios K Manatakis, Athens Naval Hospital, Greece; Nikolaos Dimitrokallis, Evangelismos General Hospital, Greece; Stylianos Kapiris, Evangelismos General Hospital, Greece; Maria Sotiropoulou, Evangelismos General Hospital, Greece; Dimitrios Stergiou, Evangelismos General Hospital, Greece; Chrysanthi Aggeli, General Hospital of Athens “G.Gennimatas “, Greece; Ioannis Triantafyllidis, General Hospital of Kavala, Greece; Iosif Chatzis, General Hospital of Thessaloniki "O Agios Dimitrios", Greece; Maria Drogouti, General Hospital of Thessaloniki "O Agios Dimitrios", Greece; Evripidis Efthymiou, General Hospital of Thessaloniki "O Agios Dimitrios", Greece; Anastasios Katsourakis, General Hospital of Thessaloniki "O Agios Dimitrios", Greece; Georgios Tzimas, Hygeia Hospital Mitera Hospital, Greece; Pavlos Koustas, Interbalkan Medical Center, Greece; Achilleas Ntinas, Interbalkan Medical Center, Greece; Alexandros Chamzin, Ippokrateio General Hospital of Athens, Greece; Maximos Frountzas, Ippokrateio General Hospital of Athens, Greece; Dimitrios Linardoutsos, Ippokrateio General Hospital of Athens, Greece; Konstantinos Toutouzas, Ippokrateio General Hospital of Athens, Greece; Dimitrios Vouros, Ippokrateio General Hospital of Athens, Greece; Alexandra Marneri, Ippokratio General Hospital Of Thessaloniki, Greece; Panagiotis Petras, Ippokratio General Hospital Of Thessaloniki, Greece; Savvas Tsaramanidis, Ippokratio General Hospital Of Thessaloniki, Greece; Spiros Delis, Konstantopouleio Hospital Athens Greece, Greece; Spyridon Davakis, Laiko General Hospital of Athens, Greece; Evangelos Felekouras, Laiko General Hospital of Athens, Greece; Lysandros Karydakis, Laiko General Hospital of Athens, Greece; Ioannis Katsaros, Laiko General Hospital of Athens, Greece; Stylianos Kykalos, Laiko General Hospital of Athens, Greece; Eleandros Kyros, Laiko General Hospital of Athens, Greece; Emmanouil Mylonakis, Laiko General Hospital of Athens, Greece; Dimitra Papalouka, Laiko General Hospital of Athens, Greece; Panagiotis Sakarellos, Laiko General Hospital of Athens, Greece; Athanasios Syllaios, Laiko General Hospital of Athens, Greece; Michail Vailas, Laiko General Hospital of Athens, Greece; Eleni Papamattheou, Metaxa Memorial Cancer Hospital of Piraeus, Greece; Ioannis Passas, Metropolitan Hospital, Greece; Christos Dervenis, Metropolitan Hospitals, Greece; Gregory G. Tsiotos, Mitera-Hygeia Hospital, Greece; Konstantinos Bramis, National and Kapodistrian University of Athens, Greece; Dimitrios Dimitroulis, National and Kapodistrian University of Athens, Greece; John Griniatsos, National and Kapodistrian University of Athens, Greece; Georgios Korovesis, National and Kapodistrian University of Athens, Greece; Panagis M Lykoudis, National and Kapodistrian University of Athens, Greece; Dimitrios Mantas, National and Kapodistrian University of Athens, Greece; Alexandros Papalampros, National and Kapodistrian University of Athens, Greece; Dimitrios Schizas, National and Kapodistrian University of Athens, Greece; Paraskevas Stamopoulos, National and Kapodistrian University of Athens, Greece; Antonios Vezakis, National and Kapodistrian University of Athens, Greece; Leonidas Chardalias, National and Kapodistrian University of Athens, Greece; Nikolaos Memos, National and Kapodistrian University of Athens, Greece; Apollon Zygomalas, Olympion General Clinic, Greece; Paraskevi Chatzikomnitsa, Papageorgiou General Hospital, Greece; Dimitris Giakoustidis, Papageorgiou General Hospital, Greece; Eleftherios Spartalis, REA MATERNITY HOSPITAL, Greece; Maria Bourazani, Saint Savvas Hospital, Greece; Dimitris P Korkolis, Saint Savvas Hospital, Greece; Panagiotis Kokoropoulos, University General Hospital Attikon, Greece; Ioannis Margaris, University General Hospital Attikon, Greece; Nikolaos V Michalopoulos, University General Hospital Attikon, Greece; Konstantinos Nastos, University General Hospital Attikon, Greece; Maria Papadoliopoulou, University General Hospital Attikon, Greece; Theodoros Sidiropoulos, University General Hospital Attikon, Greece; Konstantinos Lasithiotakis, University Hospital of Heraklion, School of Medicine, University of Crete., Greece; Grigorios Christodoulidis, University Hospital of Larissa, Greece; Konstantinos Tepetes, University Hospital of Larissa, Greece; Dimitris Zacharoulis, University Hospital of Larissa, Greece; Konstantinos Bouchagier, University Hospital Of Patras, Greece; Francesk Mulita, University Hospital Of Patras, Greece; Dimitrios Magouliotis, University of Thessaly, Greece; Nikos Sakellaridis, University of Thessaly, Greece; José Roberto Contreras, Hospital General de Enfermedades - Instituto Guatemalteco de Seguridad Social, Guatemala; Paul Bo San Lai, The Chinese University of Hong Kong, Hong Kong SAR China; Tamás Tölgyes, Uzsoki Teaching Hospital, Hungary; Karin Ulrika Johansen, Hospital of Reykjavik, Iceland; Shreeyash Modak, AIG Hospitals, India; Dharma Ram Poonia, All India Institute of Medical Sciences, India; Vaibhav Kumar Varshney, All India Institute of Medical Sciences, India; Jeewan Ram Vishnoi, All India Institute of Medical Sciences Jodhpur, India; Kunal Parasar, All India Institute of Medical Sciences, Patna, India; Monish Karunakaran, Asian Institute of Gastroenterology, India; Jenit Pareshkumar Gandhi, Care and cure multi speciality hospital, India; Smita Chauhan, Dr Ram manohar lohia institute of medical sciences, Lucknow, India; Muthukumarassamy Rajakannu, Dr Rela Institute & Medical Centre, Bharath Institute of Higher Education and Research, India; Sundeep Jain, Fortis hospital, India; Meer M Chisthi, Government Medical College Pathanamthitta, India; Arpit Bandi, Gujarat Cancer Research Institute, India; Mizelle Dsilva, Holy Family Multispeciality Hospital Bandra, India; Gaurav Sindwani, Institute of Liver and Biliary sciences (ILBS), India; Raja Kalayarasan, JIPMER, India; Naveena AN Kumar, Kasturba Medical College, Manipal Academy of Higher Education, India; Abhinav A Sonkar, King George’s Medical University, India; Ahmad Ozair, King George's Medical University, India; Shiv Rajan, King George's Medical university, India; Somnath Chattopadhyay, Kokilaben Dhirubhai Ambani Hospital, India; Debanshu Subranshu Bhaduri, M N Budhrani Cancer Institute, India; Dibyashree Paul, Medical College Kolkata, India; Srinivasan Ramachandran, Meenakshi Mission Hospital & Research Centre, India; Ramesh Ardhanari, Meenakshi Mission Hospital and Research Centre, India; Pravin Rajendra Suryawanshi, MGM Medical College & Hospital, N-6, CIDCO, India; Mayank Tripathi, MPMMCC and HBCH (unit of TMH), India; Pagadala Naga Balaji Nitesh, NRI MEDICAL COLLEGE AND SUPERSPECIALITY HOSPITAL, India; Murali Appukuttan, Pushpagiri Institute of Medical Sciences and Research Centre, India; Kashish Malhotra, Rama Medical College Hospital and Research Centre, India; Ashwin Rammohan, Rela Institute and Medical Centre, India; Mohamed Rela, Rela Institute and Medical Centre, India; Bhavin Bhupendra Vasavada, Shalby hospitals, India; Subhash Raveendran, SK Hospital, India; Sreenivasan Karuparthi, Sri Ramachandra Institute of Higher Education and Research, India; Vijay Anand Sivasubramaniam, Sri Ramakrishna specialty hospital, India; Gopi Ramu, SRM Global Hospital and Research Center, India; Rahul Gupta, Synergy Institute of Medical Sciences, India; Shraddha Patkar, Tata Memorial Hospital, India; Mahesh Goel, Tata Memorial Hospital, India; Sunil Shenvi, Trustwell hospitals, India; Harish Neelamraju Lakshmi, Yashoda Hospital, India; Dhaivat K Vaishnav, Zydus hospital, India; Ahmad Fathi Fuadi, Kariadi Hospital, Indonesia; Cosmas Rinaldi Adithya Lesmana, Mochtar Riyadi Comprehensive Cancer Center, Siloam Semanggi Hospital, Indonesia; Farzad Kakaei, Tabriz university of medical sciences, Iran; Maytham Aqeel Al-juaifari, Al-Sader Teaching Hospital, Najaf, Iraq; Ahmed Salih Aljiburi, Baghdad teaching hospital, Iraq; Hashim Talib Hashim, University of Warith Al-Anbiyaa, College of Medicine, Iraq; Abdelfatah Abdellateef Ahmed Abdelmageed, Mayo university hospital, Ireland; Anthiny Stafford, St Vincent’s University Hospital, Ireland; Kevin C Conlon, Trinity College Dublin, Ireland; Olga Esepkina, Rabin medical center, Israel; Niv Pencovich, Sheba Medical Center, Israel; Alessandro Cucchetti, Alma Mater Studiorum - University of Bologna, Italy; Francesco Moto, AOU Città della salute e della Scienza, Italy; Giacomo Deiro, AOU Città della Salute e della Scienza, “Molinette”, Italy; Edoardo Poletto, AOUI Verona, Italy; Nicola Cinardi, ARNAS Garibaldi, Italy; Paolo Ubiali, ASFO, Italy; Christian Cotsoglou, ASST Brianza - Vimercate Hospital, Italy; Stefano Granieri, ASST Brianza - Vimercate Hospital, Italy; Sissi Paleino, ASST Brianza - Vimercate Hospital, Italy; Andrea Celotti, ASST Cremona, Italy; Giovanni Ferrari, Asst Grande Ospedale Metropolitano Niguarda, Italy; Alessandro Giani, Asst Grande Ospedale Metropolitano Niguarda, Italy; Michele Mazzola, Asst Grande Ospedale Metropolitano Niguarda, Italy; Andrea Zironda, Asst Grande Ospedale Metropolitano Niguarda, Italy; Jacopo Andreuccetti, ASST Spedali Civili of Brescia, Italy; Stefano Andrianello, Aulss9, Italy; Maria Fortuna Offi, AUSL Imola, Italy; Fabrizio D'Acapito, Ausl Romagna, Morgagni-Pierantoni Hospital, Italy; Carlo Alberto Pacilio, Ausl Romagna, Morgagni-Pierantoni Hospital, Italy; Francesco Guerra, AUSL Toscana Sud Est, Italy; Elisa Bertilone, Azienda Ospedaliera Papardo, Italy; Edoardo Saladino, Azienda Ospedaliera Papardo, Italy; Giulia Deiana, Azienda Ospedaliero Universitaria di Sassari, Italy; Claudio F Feo, Azienda Ospedaliero Universitaria di Sassari, Italy; Teresa Perra, Azienda Ospedaliero Universitaria di Sassari, Italy; Alberto Porcu, Azienda Ospedaliero Universitaria di Sassari, Italy; Lapo Bencini, Azienda Ospedaliero-Universitaria Careggi, Italy; Gian Luca Grazi, Azienda Ospedaliero-Universitaria Careggi, Italy; Luca Moraldi, Azienda Ospedaliero-Universitaria Careggi, Italy; Matteo Risaliti, Azienda Ospedaliero-Universitaria Careggi, Italy; Michele Hot Ciola, Central Hospital of Bolzano/Bozen, Italy; Luca Grasso, Città della Salute e della Scienza di Torino sede Molinette, Italy; Stefano D'Ugo, Division Of Hpb Surgery - "Vito Fazzi" Hospital, Italy; Marcello Giuseppe Spampinato, Division Of Hpb Surgery - "Vito Fazzi" Hospital, Italy; Andrea Barberis, E.O. Ospedali Galliera, Italy; Emilio Bertani, European Institute of Oncology, Italy; Gianluca Rompianesi, Federico II University Hospital, Italy; Lucio Caccamo, Fond IRCCS Osp Maggiore Policlinico, Italy; Davide Citterio, Fondazione IRCCS Istituto Nazionale Tumori, Italy; Giuseppe Brisinda, Fondazione Policlinico Universitario Agostino Gemelli IRCCS, Italy; Felice Giuliante, Fondazione Policlinico Universitario Agostino Gemelli IRCCS, Italy; Giovanni Battista Levi Sandri, Fondazione Policlinico Universitario Agostino Gemelli IRCCS, Italy; Marco Maria Pascale, Fondazione Policlinico Universitario Agostino Gemelli IRCCS, Italy; Giuseppe Quero, Fondazione Policlinico Universitario Agostino Gemelli IRCCS, Italy; Angela Saviano, Fondazione Policlinico Universitario Agostino Gemelli IRCCS, Italy; Gabriele Spoletini, Fondazione Policlinico Universitario Agostino Gemelli IRCCS, Italy; Roberto Cammarata, Fondazione Policlinico Universitario Campus Bio-Medico, Italy; Tommaso Farolfi, Fondazione Policlinico Universitario Campus Bio-Medico, Italy; Tommaso Campagnaro, General and Hepatobiliary Surgery, Italy; Michele Masetti, General Surgery Unit Santa Maria Scaletta Hospital AUSL Imola, Italy; Mario Giuffrida, Guglielmo da Saliceto Hospital, Italy; Gennaro Nappo, Humanitas Research Hospital, Italy; Alessandro Zerbi, Humanitas Research Hospital, Italy; Gianluca Garulli, Infermi Rimini, Italy; Matteo Serenari, IRCCS Azienda Ospedaliero-Universitaria di Bologna, Italy; Marco Cereda, IRCCS Foundation San Gerardo dei Tintori, Italy; Luca Gianotti, IRCCS Foundation San Gerardo dei Tintori, Italy; Sara Massironi, IRCCS Foundation San Gerardo dei Tintori, Italy; Fabio Uggeri, IRCCS Foundation San Gerardo dei Tintori, Italy; Salvatore Gruttadauria, IRCCS ISMETT UPMC Italy, Italy; Alberto Aiolfi, IRCCS Ospedale Galeazzi - Sant'Ambrogio, Italy; Diego Coletta, IRCCS Regina Elena National Cancer Institute, Italy; Massimo Falconi, IRCCS San Raffaele Hospital, Vita-Salute University, Italy; Alice Frontali, IRCCS San Raffaele Hospital, Vita-Salute University, Italy; Luca Ottaviani, IRCCS San Raffaele Hospital, Vita-Salute University, Italy; Andrea Belli, Istituto Nazionale Tumori - IRCCS - Fondazione G. Pascale, Italy; Renato Patrone, Istituto Nazionale Tumori - IRCCS - Fondazione G. Pascale, Italy; Marco Brizzolari, IV Chirurgia, Ospedale Di Treviso; Università Di Padova, Italy; Simone Novello, IV Chirurgia, Ospedale Di Treviso; Università Di Padova, Italy; Tommaso Stecca, IV Chirurgia, Ospedale Di Treviso; Università Di Padova, Italy; Giacomo Zanus, IV Chirurgia, Ospedale Di Treviso; Università Di Padova, Italy; Irene Urciuoli, La Sapienza, Italy; Salomone Di Saverio, Madonna del Soccorso Hospital AST Ascoli Piceno, Italy; Ilenia Merlini, Madonna del Soccorso Hospital AST Ascoli Piceno, Italy; Marco Vivarelli, Marche Polytechnic University, Italy; Vincenzo Neri, Medical School --- University of Foggia, Italy; Giuseppe Giuliani, Misericordia Hospital, Italy; Michele Tedeschi, Miulli Hospital, Italy; Leonardo Vincenti, National Institute of Gastroenterology “S. De Bellis”, IRCCS Research Hospital, Italy; Vincenzo Papagni, National Institute of Gastroenterology, Saverio de Bellis Research Hospital, Italy; Giorgio Querini, Ospedale "Castelli", Verbania - ASL VCO, Italy; Daniele Ferraro, Ospedale Antonio Cardarelli, Italy; Donatella Pisaniello, Ospedale Antonio Cardarelli, Italy; Alfonso Giovanni Recordare, Ospedale dell'Angelo Venezia Mestre, Italy; Serena Langella, Ospedale Mauriziano, Italy; Giuseppe Barbato, Ospedale San Giovanni di Dio, Italy; Linda Gabellini, Ospedale San Giovanni di Dio, Italy; Matteo Santoliquido, Ospedale San Martino, Italy; Stefano Scabini, Ospedale San Martino, Italy; Carlo Gazia, Ospedale San Pietro Fatebenefratelli, Italy; Marco Palucci, Ospedale Santo Spirito, Italy; Alessandro Iacomino, Ospedale SS Giovanni e Paolo - Venezia, Italy; Paolo Pizzini, Papa Giovanni XXIII Hospital, Italy; Mario Giuffrida, Parma University Hospital, Italy; Giovanni Butturini, Pederzoli Hospital, Italy; Paolo Regi, Pederzoli Hospital, Italy; Isabella Frigerio, Pederzoli Hospital, Italy; Mohammad Abu Hilal, Poliambulanza Hospital, Italy; Giovanna Di Meo, Policlinico Di Bari, Italy; Angela Gurrado, Policlinico Di Bari, Italy; Mario Testini, Policlinico Di Bari, Italy; Enrico Pinotti, Policlinico San Pietro, Italy; Mauro Montuori, Policlinico San Pietro, Italy; Valentina Murzi, Policlinico Universitario di Cagliari, Italy; Ahmed Mohammed Farid Mahmoud Mansour, Politecnico di Torino, Italy; Davide Papis, S. Anna Hospital, Italy; Sara Napetti, San Bortolo Hospital, Italy; Alberto Brolese, Santa Chiara Hospital - APSS, Italy; Stefano Marcucci, Santa Chiara Hospital - APSS, Italy; Giorgio Giraudo, Santa Croce And Carle Hospital, Italy; Diego Sasia, Santa Croce And Carle Hospital, Italy; Davide Papis, Sant'Anna Hospital, Italy; Alessandro Coppola, Sapienza University of Rome, Italy; Fabio Melandro, Sapienza University of Rome, Italy; Niccolo Petrucciani, Sapienza University of Rome, Italy; Sarah Molfino, Spedali Civili di Brescia, Italy; Alberto Balduzzi, The Pancreas Institute, University of Verona, Italy; Matteo De Pastena, The Pancreas Institute, University of Verona, Italy; Giuseppe Malleo, The Pancreas Institute, University of Verona, Italy; Roberta Angelico, Tor Vergata University of Rome, Italy; Luigi Eduardo Conte, Tor Vergata University of Rome, Italy; Tommaso Maria Manzia, Tor Vergata University of Rome, Italy; Marco Pellicciaro, Tor Vergata University of Rome, Italy; Luca Toti, Tor Vergata University of Rome, Italy; Giovanni Pirozzolo, ULSS3 Serenissima, Italy; Elio Jovine, University of Bologna IRCCS Sant’Orsola, Italy; Riccardo Casadei, University of Bologna, Italy; Gian Luca Baiocchi, University of Brescia, Italy; Mauro Podda, University of Cagliari, Italy; Isidoro Di Carlo, University of Catania, Italy; Adriana Toro, University of Catania, Italy; Marcello Di Martino, University of Eastern Piedmont - Ospedale Maggiore, Italy; Luca Mastracci, University of Genova, Italy; Roberto Ballarin, University of Modena, Italy; Franco Lumachi, University of Padova, Italy; Giovanni Marchegiani, University of Padova, Italy; Anna Caterina Milanetto, University of Padova, Italy; Lucia Moletta, University of Padova, Italy; Giampaolo Perri, University of Padova, Italy; Salvatore Buscemi, University of Palermo, Italy; Francesco Ferrara, University of Palermo, Italy; Lorenzo Cobianchi, University of Pavia, Italy; Roberto Cirocchi, University of Perugia, Italy; Ugo Boggi, University of Pisa, Italy; Annalisa Comandatore, University of Pisa, Italy; Gregorio Di Franco, University of Pisa, Italy; Niccolò Furbetta, University of Pisa, Italy; Emanuele Federico Kauffmann, University of Pisa, Italy; Luca Morelli, University of Pisa, Italy; Niccolò Napoli, University of Pisa, Italy; Mario Gruppo, Veneto Institute Of Oncology IOV – IRCCS, Italy; Toru Nakamura, Hokkaido University, Japan; Ryoichi Miyamoto, Ibaraki Medical Center, Tokyo Medical University, Japan; Akio Saiura, Juntendo University, Japan; Keiichi Okano, Kagawa University, Japan; Shintaro Yagi, Kanazawa University, Japan; Daisuke Hashimoto, Kansai Medical University, Japan; Masayuki Tanaka, Keio University School of Medicine, Japan; Ippei Matsumoto, Kindai University Faculty of Medicine, Japan; Hiromitsu Maehira, Shiga University of Medical Science, Japan; Keiichi Akahoshi, Tokyo Medical and Dental University, Japan; Yuichi Nagakawa, Tokyo Medical University, Japan; Almu'atasim Khamees, -, Jordan; Mahmoud Rawhi Mahafdah, Irbid Speciality Hospital, Jordan; Mohammad Alzoubi, Jordan University Hospital, Jordan; Auman Obed, Jordan University Hospital, Jordan; Mohammad A. Albatayneh, Jordan University of Science and Technology, Jordan; Khaled Ali Obeidat, Jordan University of Science and Technology, Jordan; Khaled Zaitoun, Jordan University of Science and Technology, Jordan; Amr A. Al Hammoud, King Abdullah University Hospital, Jordan; Raghad Alhammoud, King Abdullah University Hospital, Jordan; Ildar Fakhradiyev, Kazakh National Medical University, Kazakhstan; Zhanat Spayayev, National Research Oncology Centre, Kazakhstan; Bekkozha Yeskendirov, National Scientific Center for Oncology and Transplantation, Kazakhstan; Chokhan Aitbayev, NNOC, Kazakhstan; Karan Gandhi, MP Shah Hospital, Kenya; Haralds Plaudis, Riga East Clinical University Hospital, Latvia; Mohammad Jawad Khalife, AUBMC, Lebanon; Marwa Mohammed Morgom, Alestiqlal hospital, Libya; Elham Hussin Bareig, Aljala, Libya; Fras Ammar D Elhajdawe, Metiga Hospital, Libya; Abdulhadi Alshatshat, National Cancer Institute, Libya; Reem Jamal Ghmagh, Tripoli Central Hospital, Libya; Mohamed Alsori Alharari, Tripoli University Hospital, Libya; Dua Mansour Ali, Tripoli University Hospital, Libya; Yara Talal Bariun, Tripoli University Hospital, Libya; Tasneem Musbah Faraj, Tripoli University Hospital, Libya; Ahmed Msherghi, University of Tripoli, Libya; Najat Shaban Salem Ben Hasan, Zliten medical center, Libya; Jonas Jurgaitis, Klaipeda University Hospital, Lithuania; Giedrius Barauskas, Lithuanian University of Health Sciences, Lithuania; Povilas Ignatavicius, Lithuanian University of Health Sciences, Lithuania; Tomas Vanagas, Lithuanian University of Health Sciences, Lithuania; Mindaugas Kvietkauskas, Vilnius University Hospital Santaros Klinikos, Lithuania; Andee Dzulkarnaen Zakaria, School of Medical Sciences & Hospital Universiti Sains Malaysia, Malaysia; Krishnan Raman, Sime Darby Medical Centre, Malaysia; Boon Koon Yoong, Sunway Medical Centre, Malaysia; Azlanudin Azman, Universiti Kebangsaan Malaysia Medical Centre, Malaysia; Peng Soon Koh, Universiti Malaya, Malaysia; Carlos Florez Zorrilla, Hospital Valentin Gomez Farias-ISSSTE, Mexico; Miguel A Mercado, Inst Nal de la Nutrición Salvador Zubiran, Mexico; Juan Sebastián García-Herrera, Instituto Nacional Cancerologia Mexico, Mexico; Gerardo Miranda-Devora, Instituto Nacional Cancerologia Mexico, Mexico; Alejandro Eduardo Padilla Rosciano, Instituto Nacional Cancerologia Mexico, Mexico; Eugen Tcaciuc, Nicolae Testemitanu State University of Medicine and Pharmacy, Moldova; Svetlana Turcan, Nicolae Testemitanu State University of Medicine and Pharmacy, Moldova; Sarnai Erdene, Mongolian National University of Medical Sciences, Mongolia; Amina Houmada, CHU Ibn Sina, Morocco; Bounaim Ahmed, Military Universitaire Hospital Med V Rabat Morocco, Morocco; Sidi Mohammed Bouchentouf, Military Universitaire Hospital Med V Rabat Morocco, Morocco; Imad El Azzaoui, Military Universitaire Hospital Med V Rabat Morocco, Morocco; Abdelilah Ghannam, Military Universitaire Hospital Med V Rabat Morocco, Morocco; Amine Benkabbou, National Institute Of Oncology, Mohammed V University In Rabat, Morocco; Oumayma Lahnaoui, National Institute Of Oncology, Mohammed V University In Rabat, Morocco; Mohammed Anass Majbar, National Institute Of Oncology, Mohammed V University In Rabat, Morocco; Amine Souadka, National Institute Of Oncology, Mohammed V University In Rabat, Morocco; Pueya Rashid Nashidengo, Windhoek Central Hospital, Namibia; Francis William Quayson, Windhoek Central Hospital, Namibia; John Tabiri Abebrese, Windhoek Central Hospital, Namibia; Roshan Ghimire, Kathmandu Medical College Teaching Hospital, Nepal; Dhiresh Kumar Maharjan, Kathmandu Medical College Teaching Hospital, Nepal; Paleswan Joshi Lakhey, Tribhuvan University Teaching Hospital, Nepal; Marc G Besselink, Amsterdam UMC, Netherlands; Pim Bastiaan Olthof, University Medical Center Groningen, Netherlands; Vincent Erwin de Meijer, University of Groningen and University Medical Center Groningen, Netherlands; Louise Barbier, Auckland City Hospital, New Zealand; Michael Jen Jie Chu, Laparoscopy Auckland, New Zealand; Sanket Srinivasa, North Shore Hospital, New Zealand; Jonathan Koea, University of Auckland, New Zealand; John Albert Windsor, University of Auckland, New Zealand; Ibrahim Umar Garzali, Aminu Kano Teaching Hospital, Nigeria; Abubakar Bala Muhammad, Aminu Kano Teaching Hospital, Nigeria; Esteem Tagar, Irrua Specialist Teaching Hospital, Nigeria; Olusegun Isaac Alatise, Obafemi Awolowo University, Nigeria; Omorodion Omoruyi Irowa, University of Benin/ University of Benin Teaching Hospital, Nigeria; Knut Jørgen Labori, Oslo University Hospital, Rikshospitalet, Norway; Kjetil Soreide, Stavanger University Hospital, Norway; Nooreen Yousuf Al Balushi, The Royal Hospital, Oman; Zaigham Abbas, Dr. Ziauddin University Hospital Clifton, Pakistan; Muhammad Imran Khokhar, Gujranwala Medical College, Pakistan; Belinda Sanchez, H. Regional Universitario de Málaga, Pakistan; Syed Muhammad Shafqatullah, Jinnah Postgraduate Medical Center, Pakistan; Naeem Ahmed Butt, Midland Doctors, Pakistan; Abu Bakar Hafeez Bhatti, Shifa International Hospital Islamabad Pakistan, Pakistan; Uzma Soomro, Sindh Institute of Urology and Transplantation, Pakistan; Ahmad Musleh Abu Yousef, Al-Ahli Hospital-Hebron-Palestine, Palestinian Territories; Mohammad Issa Sowad, Al-Ahli Hospital-Hebron-Palestine, Palestinian Territories; Jalal Ahmad Taqatqa, Al-Ahli Hospital-Hebron-Palestine, Palestinian Territories; Qusai Nimer Zreqat, Al-Ahli Hospital-Hebron-Palestine, Palestinian Territories; Emilio Domingo Tonina, Hospital de Especialidades Quirurgicas "Ingavi", Paraguay; Luis Poggi, British American Hospital, Peru; Rafael Garatea Grau, Clínica Ricardo Palma, Peru; Juan Carlos Marcos Enriquez, Hospital Nacional Edgardo Rebagliati Martins, Peru; Jose L. Paz-Ibarra, Hospital Nacional Edgardo Rebagliati Martins, Peru; Cenon Reynoso Alfonso, Ateneo de Manila University School of Medicine and Public Health, Philippines; Catherine Sia Cheng Teh, Makati Medical Center, Philippines; Piotr Major, Jagiellonian University Medical College, Poland; Maciej Matyja, Jagiellonian University Medical College, Poland; Marek Sierzega, Jagiellonian University Medical College, Poland; Oskar Kornasiewicz, Medical University of Warsaw, Poland; Marek Wroński, Medical University of Warsaw, Poland; Krzysztof Zieniewicz, Medical University of Warsaw, Poland; Krzysztof Grzegorz Jeziorski, National Institute of Geriatrics, Rheumatology and Rehabilitation, Poland; Justyna Wyroslak-Najs, University Teaching Hospital No1, Poland; Nádia Tenreiro, Centro Hospitalar de Trás-os-Montes e Alto Douro, Portugal; Henrique Alexandrino, Coimbra University Hospital Center, Portugal; Emanuel Vigia, Hospital de Curry Cabral - Centro Hospitalar Universitário de Lisboa Central, Portugal; Paulo Soares Mira, Hospital Prof. Dr. Fernando Fonseca, Portugal; Ionut Negoi, Carol Davila University of Medicine and Pharmacy Bucharest, Emergency Hospital of Bucharest, Romania; Matei Bratu, Emergency Clinical Hospital of Bucharest, Romania; Adrian Ioan Popentiu, Emergency Military Hospital, Romania; Nicolae Bacalbasa, Fundeni Clinical Institute, Romania; Traian Dumitrascu, Fundeni Clinical Institute, Romania; Sorin Traian Barbu, Iuliu Hatieganu University Of Medicine And Pharmacy, Romania; Dana Monica Iancu, Iuliu Hatieganu University Of Medicine And Pharmacy, Romania; Octav Ginghina, Oncological Institute Bucharest, Romania; Mara Mardare, Oncological Institute Bucharest, Romania; Raluca Bodea, Regional Institute of Gastroenterology and Hepatology, Romania; Gabriel Mihail Dimofte, Regional Institute of Oncology, Romania; Sorinel Lunca, Regional Institute of Oncology, Romania; Stefan Morarasu, Regional Institute of Oncology, Romania; Ana-Maria Musina, Regional Institute of Oncology, Romania; Iulian Brezean, Spitalul Clinic Dr. I. Cantacuzino, Romania; Eduard Lucian Catrina, Spitalul Clinic Dr. I. Cantacuzino, Romania; Sorin Petrea, Spitalul Clinic Dr. I. Cantacuzino, Romania; Alina Tantau, The 4 Medical Clinic, Romania; Dmitry Akhaladze, Dmitry Rogachev National Medical Research Center Of Pediatric Hematology, Oncology and Immunology, Russia; Igor Khatkov, Moscow Clinical Scientific Center Named After A.S. Loginov, Russia; Pavel Tyutyunnik, Moscow Clinical Scientific Center Named After A.S. Loginov, Russia; Vasiliy Ivanovich Davydkin, National Research Mordovian State University, Russia; Denis Mizgirev, Northern State Medical University, Russia; Nikolay Bagmet, Petrovsky National Research Center of Surgery, Russia; Vladimir Evgenievich Zagainov, Privolzhsky Research Medical University, Russia; Evgeniy Drozdov, Siberian State Medical University, Russia; Mohammed Alharthi, King Abdulaziz University Hospital, Saudi Arabia; Murad Aljiffry, King Abdulaziz University Hospital, Saudi Arabia; Mohammed Ghunaim, King Abdulaziz University Hospital, Saudi Arabia; Mohammad A. Al Hamoud, WeCare Hospital, Saudi Arabia; Ibrahima Sall, Military teaching hospital, Senegal; Marko Dragoslav Bogdanovic, Clinic for Digestive Surgery, Serbia; Igor Ignjatovic, Clinical Center Of Serbia, Serbia; Aleksandar Radoslav Karamarkovic, Faculty of Medicine University of Belgrade, Serbia; Dejan Radenkovic, Faculty of Medicine, University of Belgrade, Serbia; Mihailo Bezmarević, Military Medical Academy, Serbia; Aleksandar Bogdanovic, University Clinical Center of Serbia, Serbia; Danijel Galun, University Clinical Center of Serbia, Serbia; Marko Zivanovic, University Clinical Center of Serbia, Serbia; Vladica Velibor Cuk, Zvezdara University Medical Center, Serbia; Jovan Todor Juloski, Zvezdara University Medical Center, Serbia; Aleksandar Karamarkovic, Zvezdara University Medical Center, Serbia; Ljiljana Velibor Milic, Zvezdara University Medical Center, Serbia; Alfred Kow, National University Hospital Singapore, Singapore; Brian K. P. Goh, Singapore General Hospital, Singapore; Vishal G Shelat, Tan Tock Seng Hospital, Singapore; Arpad Ivanecz, University Medical Center Maribor, Slovenia; John Wesley Samuel Devar, Chris Hani Baragwanath Academic Hospital/University Of The Witwatersrand, South Africa; Zafar Ahmed Khan, Chris Hani Baragwanath Academic Hospital/University Of The Witwatersrand, South Africa; Jones A. O. Omoshoro-Jones, Chris Hani Baragwanath Academic Hospital/University Of The Witwatersrand, South Africa; Martin Derrick Smith, Chris Hani Baragwanath Academic Hospital/University Of The Witwatersrand, South Africa; Zain Ally, Helen Joseph Hospital WITS, South Africa; Mark John Victor Hewat, Kingsbury Hospital, South Africa; Neha Kumar, Sefako Makgatho Health Sciences University, South Africa; Imraan Sardiwalla, Sefako Makgatho Health Sciences University, South Africa; Modise Zacharia Koto, Sefako Makgatho University, South Africa; Andries Bert Steenkamp, Stellenbosch and private practice, South Africa; Stefan Hofmeyr, Stellenbosch University, South Africa; Muaz Ahmed, Steve Biko Academic Hospital, South Africa; Wanga Mtimkulu, University of Cape Town, South Africa; Colin Byron Noel, Universitas Academic Hospital, South Africa; Eduard Jonas, University of Cape Town, South Africa; Sanju Sobnach, University of Cape Town, South Africa; Christo Kloppers, University of Cape Town / Groote Schuur Hospital, South Africa; Peter Barrow, Wits DGMC, South Africa; Iago Justo, "12 de Octubre" University Hospital, Spain; Juli Busquets, Bellvitge University Hospital, Spain; Mikel Prieto Calvo, Cruces University Hospital, Spain; Fabio Ausania, Hospital Clinic, IDIBAPS, University of Barcelona, Spain; Filippo Landi, Hospital Clinic, IDIBAPS, University of Barcelona, Spain; Sofia de la Serna, Hospital Clínico San Carlos, Spain; Jana Dziakova, Hospital Clínico San Carlos, Spain; María Elia Pérez-Aguirre, Hospital Clínico San Carlos, Spain; Marina Garcés-Albir, Hospital Clínico Universitario de Valencia, Spain; Mario Rodriguez-Lopez, Hospital Clínico Universitario de Valladolid, Spain; Mario Montes Manrique, Hospital Clínico Universitario de Valladolid, Spain; Santiago Sánchez-Cabús, Hospital de la Santa Creu i Sant Pau, Spain; Benedetto Ielpo, Hospital del Mar, Spain; Jose M Ramia, Hospital General Universitario de Alicante Dr. Balmis, Spain; Esther Pilar García Santos, Hospital General Universitario de Ciudad Real, Spain; David Padilla-Valverde, Hospital General Universitario de Ciudad Real, Spain; Francisco Espin, Hospital Germans Trias i Pujol, Spain; Jordi Navinés-López, Hospital Germans Trias i Pujol, Spain; Mariana González Zunzarren, Hospital Infanta Sofia, Spain; Jose Carlos Rodriguez Pino, Hospital Son Espases, Spain; Pablo Muriel Alvarez, Hospital Universitari Arnau de Vilanova, Spain; Maria Teresa Albiol, Hospital Universitari de Girona Dr. Josep Trueta, Spain; Ernest Castro, Hospital Universitari de Girona Dr. Josep Trueta, Spain; Santiago Lopez-Ben, Hospital Universitari de Girona Dr. Josep Trueta, Spain; Maria Isabel Garcia-Domingo, Hospital Universitari Mútua de Terrassa, Spain; Ramón Soliva Domínguez, Hospital Universitari Sagrat Cor, Spain; Jaime López-Sánchez, Hospital Universitario de Salamanca, Universidad de Salamanca, Spain; Rosa Jorba, Hospital Universitari de Tarragona Joan XXIII, Spain; Manuel Marcello, Hospital Universitario Fundación Alcorcón, Spain; Esteban Cugat Andorrá, Hospital Universitario Germans Trias i Pujol, Spain; Eva María Montalvá, Hospital Universitario La Fe, Spain; Elena Martin-Perez, Hospital Universitario La Princesa, Spain; Maria del Mar Rico, Hospital Universitario Torrecárdenas, Spain; Mario Serradilla-Martín, Hospital Universitario Virgen de las Nieves, Spain; Luis Barneo, Hospital Universitario Central de Asturias, Spain; Irene Ortega Vázquez, Infanta Sofia University Hospital, Spain; Alejandro Serrablo, Miguel Servet University Hospital, Spain; Carmen Cepeda-Franco, Virgen del Rocío University Hospital, Spain; Javier Mínguez, University Hospital Príncipe de Asturias, Spain; Miguel Ángel Suarez-Muñoz, University Hospital Virgen de la Victoria, Spain; Dario Martinez-Baena, Valme University Hospital, Spain; Pablo Parra-Membrives, Valme University Hospital, Spain; Luis Tallon-Aguilar, Virgen del Rocío University Hospital, Spain; Kithsiri Janakantha Senanayake, Faculty of Medicine and Allied Health Sciences, Sri Lanka; Duminda Subasinghe, University of Colombo, Sri Lanka; Joel Arudchelvam, University of Colombo, Sri Lanka; Khadijah Ali Abdalrahman Hamad, Alneelain University, Sudan; Thuraya Mohammed Elbola, Elmanagil Teaching Hospital, Sudan; Ahmed Mohamed Ibrahim Mohamed, Gadarif Teaching Hospital, Sudan; Ioannis Gkekas, Department of Surgery and Perioperative Science, Sweden; Jennie Engstrand, Karolinska Institutet, Sweden; Poya Ghorbani, Karolinska Institutet, Sweden; Ernesto Sparrelid, Karolinska Institutet, Sweden; Roland Gert Andersson, Lund University, Sweden; Bodil Andersson, Lund University, Sweden; Bobby Tingstedt, Lund University, Sweden; Svein Olav Bratlie, Sahlgrenska University Hospital, Sweden; Urban Arnelo, Umeå University, Sweden; Roberto Valente, Umeå University, Sweden; Chiara Maria Scandavini, Umeå University, Sweden; Asif Halimi, Umeå University Hospital, Sweden; Ira Sotirova, Umeå University Hospital, Sweden; Dennis Björk, University Hospital Linköping, Sweden; Thomas Rikard Gasslander, University Hospital Linköping, Sweden; Per Sandström, University Hospital Linköping, Sweden; Sofia Xenaki, BAG Bundesamt für Gesundheit Schweiz, Switzerland; Pascal Probst, Cantonal Hospital Thurgau, Switzerland; Lukasz Filip Grochola, Cantonal Hospital Winterthur, Switzerland; Christoph Kuemmerli, Clarunis, University Centre for Gastrointestinal and Liver Diseases, St. Clara Hospital and University Hospital, Switzerland; Joël L. Lavanchy, Clarunis, University Centre for Gastrointestinal and Liver Diseases, St. Clara Hospital and University Hospital, Switzerland; Christian Eugen Oberkofler, Hirslanden Clinics Zurich, Switzerland; Jan Schmidt, Hirslanden Clinics Zurich, Switzerland; Beat Moeckli, Hôpitaux Universitaires de Genève, Switzerland; Andrea Peloso, Hôpitaux Universitaires de Genève, Switzerland; Frederic Ris, Hôpitaux Universitaires de Genève, Switzerland; Emmanuel Melloul, Lausanne University Hospital, Switzerland; Stefan Gutknecht, Triemli Hospital Zurich, Switzerland; Beat Gloor, University of Bern, Inselspital, Switzerland; Dominique Lisa Birrer, University Hospital Zürich, Switzerland; Hasan Nabil Al Houri, Al Assad University Hospital, Syria; Ahmad Nabil Alhouri, Al Assad University Hospital, Syria; Ahmad Amir Kayali, University of Aleppo, Syria; Ming-Chin Yu, New Taipei Municipal Hospital, Chang-Gung Medical Foundation, Taiwan; Shamir O. Cawich, University of the West Indies, Trinidad & Tobago; Ali Kchaou, Habib Bourguiba University Hospital, Tunisia; Oussama Baraket, Hôpital Habib Bougatfa, Tunisia; Karim Ben Hamida, Hospital of the Internal Security Forces of La Marsa, Tunisia; Ahmed Ben Mahmoud, La Rabta Hospital, Tunisia; Chaima Yakoubi, La Rabta Hospital, Tunisia; Houssem Ammar, Sahloul Hospital, Tunisia; Mohamed Ben Mabrouk, Sahloul Hospital, Tunisia; Mohamed Amine Said, Sahloul Hospital, Tunisia; Fahri Hüseyin Martlı, Ankara Bilkent City Hospital, Turkey; Mesut Tez, Ankara City Hospital, Turkey; Fatih Aslan, Ankara Etlik City Hospital, Turkey; Ferit Aydın, Ankara Etlik City Research and Education Hospital, Turkey; Elvan Onur Kirimker, Ankara University, Turkey; Kaan Karayalcin, Ankara University Medical School, Turkey; Direnç Yiğit, Antalya Private OFM Hospital, Turkey; Vusal Aliyev, Bağcılar Medilife Hospital, Turkey; Alpen Yahya Gümüşoğlu, Bakırköy Dr. Sadi Konuk Research and Training Hospital, Turkey; Hamit Ahmet Kabuli, Bakırköy Dr. Sadi Konuk Research and Training Hospital, Turkey; Mustafa Yener Uzunoglu, Bursa City Hospital, Turkey; Nidal Iflazoglu, Bursa City Hospital, Turkey; Hanife Seyda Ulgur Genc, Cihanbeyli State Hospital, Turkey; Ulaş Aday, Dicle University School of Medicine, Turkey; Omer Yalkin, Doruk Nilufer Hospital, Turkey; Ahmet Çoker, Ege University, Turkey; Kursat Dikmen, Gazi University, Turkey; Sevket Baris Morkavuk, Gülhane Training and Research Hospital, Turkey; Arda Isik, Istanbul Medeniyet University, Turkey; Pırıltı Özcan, Istanbul University Faculty of Medicine (Çapa), Turkey; Ali Cihat Yildirim, Kütahya Health Sciences University, Turkey; Ali Emre Atici, Marmara University, Turkey; Ilhan Karabicak, MedicalPark Hospital, Turkey; Koray Topgül, Medicana Ataşehir Hospital, Turkey; Murat Dayangac, Medipol University Hospital, Turkey; Osman Abbasoglu, private, Turkey; Erhan Eröz, Sakarya Education and Research Hospital, Turkey; Ibrahim Furkan Küçük, Sakarya Training and Research Hospital, Turkey; Zulfu Bayhan, Sakarya University Faculty of Medicine, Turkey; Emre Gonullu, Sakarya University Faculty of Medicine, Turkey; Ahmet Tarik Harmantepe, Sakarya University Training and Research Hospital, Turkey; Elif Colak, Samsun Training and Research Hospital, Turkey; Gulcin Ercan, Sultan Abdülhamid Han Training and Research Hospital, Turkey; Murat Kalın, Sultan Abdülhamid Han Training and Research Hospital, Turkey; Haluk Kerim Karakullukcu, Sultan Abdülhamid Han Training and Research Hospital, Turkey; Omer Faruk Ozkan, Sultan Abdülhamid Han Training and Research Hospital, Turkey; Mahmut Onur Kültüroğlu, Surgical Oncology, Turkey; Acar Tuzuner, TOBB ETÜ University, Memorial Ankara, Turkey; Özgül Düzgün, Ümraniye Training and Research Hospital, Turkey; Emre Furkan Kirkan, Ümraniye Training and Research Hospital, Turkey; Andriy Kozachenko, Kharkiv Regional Center of Oncology, Ukraine; Valeriia Sumarokova, Kyiv City Clinical Oncology Center, Ukraine; Serge Chooklin, Lviv Regional Clinical Hospital, Ukraine; Dmytro Cheverdiuk, National Cancer Institute, Ukraine; Kostiantyn Kopchak, National Cancer Institute, Ukraine; Viacheslav Kopetskyi, National Cancer Institute, Ukraine; Oleksandr Oleksandrovich Kvasivka, National Cancer Institute, Ukraine; Andrii Volodymyrovych Klymenko, Zaporizhzhya State Medical and Pharmaceutical University, Ukraine; Solomon Kuruvilla John, Sheikh Khalifa Hospital, United Arab Emirates; Oktay Irkorucu, University of Sharjah, College of Medicine, United Arab Emirates; Raaj Kumar Praseedom, Cambridge University Hospital Foundation NHS Trust, United Kingdom; Anita Balakrishnan, Cambridge University Hospitals NHS Foundation Trust, United Kingdom; Mohamed Bekheit, East Lancashire Hospitals NHS Trust, United Kingdom; Georgios Ioannis Verras, Epsom and St Helier University Hospitals NHS Trust, United Kingdom; Rohan Thakkar, Freeman Hospital, United Kingdom; Reena Ravikumar, HCA Healthcare UK, United Kingdom; Swathikan Chidambaram, Imperial College London, United Kingdom; Andrea Frilling, Imperial College London, United Kingdom; Munir Tarazi, Imperial College London, United Kingdom; Dimitrios Oikonomou, King’s College Hospital, United Kingdom; John David Isherwood, Leicester HPB Department, United Kingdom; Jenifer Barrie, Manchester Royal Infirmary, University of Manchester, United Kingdom; Nicola de’Liguori Carino, Manchester Royal Infirmary, University of Manchester, United Kingdom; Georgios Gemenetzis, Ninewells Hospital and Medical School, United Kingdom; Gordon Gregory, Nottingham University Hospitals, United Kingdom; Tomas Urbonas, Oxford University Hospitals NHS Foundation Trust, United Kingdom; Alex Nicolas Gordon-Weeks, Oxford University Hospitals NHS Foundation Trust, United Kingdom; Rumyana Rumenova Smilevska, Oxford University Hospitals NHS Foundation Trust, United Kingdom; Anas Mardini, Princess Royal University Hospital, United Kingdom; Nikolaos Chatzizacharias, Queen Elizabeth Hospital, United Kingdom; Bobby V. M. Dasari, Queen Elizabeth Hospital, United Kingdom; Michael Feretis, Queen Elizabeth Hospital, United Kingdom; Peter Lawrence Zaki Labib, Queen Elizabeth Hospital Birmingham, United Kingdom; Bulathsinhalage K. Sandun Bulathsinhala, Royal Free London NHS Foundation Trust, United Kingdom; Hewa Malavige Sasindu Sudara De Silva, Royal Free London NHS Foundation Trust, United Kingdom; Giuseppe Kito Fusai, Royal Free London NHS Foundation Trust, United Kingdom; Upul Gonapinuwala, Royal Free London NHS Foundation Trust, United Kingdom; Christopher Hadjittofi, Royal Free London NHS Foundation Trust, United Kingdom; Manikandan Kathirvel, Royal Free London NHS Foundation Trust, United Kingdom; Ioannis D. Kostakis, Royal Free London NHS Foundation Trust, United Kingdom; David Nasralla, Royal Free London NHS Foundation Trust, United Kingdom; Stephanos Pericleous, Royal Free London NHS Foundation Trust, United Kingdom; Theodora Pissanou, Royal Free London NHS Foundation Trust, United Kingdom; Sebastian Manuel Staubli, Royal Free London NHS Foundation Trust, United Kingdom; Pascale Marie Tinguely, Royal Free London NHS Foundation Trust, United Kingdom; Navneet Tiwari, Royal Free London NHS Foundation Trust, United Kingdom; Ewen M. Harrison, Royal Infirmary of Edinburgh, United Kingdom; Rowan Parks, Royal Infirmary of Edinburgh, United Kingdom; Stephen J. Wigmore, Royal Infirmary of Edinburgh, United Kingdom; Adam Frampton, Royal Surrey NHS Foundation Trust, United Kingdom; Antonios Athanasiou, Royal Sussex County Hospital, United Kingdom; Alastair Young, St James’s University Hospital, United Kingdom; Ricky Harminder Bhogal, The Royal Marsden Hospital, United Kingdom; Muhammad Harris Siddique, University Hospitals of North Midlands (UHNM), United Kingdom; Francis P. Robertson, University Hospitals of Leicester, United Kingdom; Giuseppe Garcea, University Hospitals of Leicester, United Kingdom; Tareq Al Saoudi, University Hospitals of Leicester NHS Trust, United Kingdom; Deep J. Malde, University Hospitals of Leicester NHS Trust, United Kingdom; Camila Hidalgo Salinas, Wellington Hospital, HCA, United Kingdom; Amjad Khalil, Wellington Hospital, HCA, United Kingdom; Iswanto Sucandy, AdventHealth Tampa, United States; Andrei Cocieru, Akron City Hospital, United States; Mark Fairweather, Brigham and Women's Hospital/Harvard Medical School, United States; Cristina Ferrone, Cedars-Sinai, United States; Laleh Melstrom, City of Hope National Medical Center, United States; Zoe Garoufalia, Cleveland Clinic Florida, United States; Mayank Roy, Cleveland Clinic Florida, United States; John A. Chabot, Columbia University, United States; Michael D. Kluger, Columbia University, United States; Beth A. Schrope, Columbia University, United States; Shishir Kumar Maithel, Emory University, United States; Ernest E. Moore, Ernest E. Moore Shock Trauma Center at Denver Health, United States; Jin He, Johns Hopkins University, United States; Ahmed Dehal, Kaiser Permanente School of Medicine, United States; Alice C. Wei, Memorial Sloan Kettering Cancer Center, United States; Myron Schwartz, Mount Sinai, United States; Akhil Chawla, Northwestern University, United States; David M. Heimann, NY Harbor Healthcare System, United States; Michael G. Wayne, NYP Brooklyn Methodist Hospital, United States; D. Brock Hewitt, NYU Grossman School of Medicine, United States; Katherine A. Morgan, NYU Grossman School of Medicine, United States; Flavio G. Rocha, Oregon Health and Science University, United States; Kerri Ann Simo, ProMedica Health System, United States; Brian Richard Davis, Texas Tech Paul L. Foster School of Medicine, United States; Diamantis I. Tsilimigras, The Ohio State University Wexner Medical Center, United States; Wilbur Bowen Bowne, Thomas Jefferson University, United States; Harish Lavu, Thomas Jefferson University, United States; Avinoam Nevler, Thomas Jefferson University, United States; Charles John Yeo, Thomas Jefferson University, United States; Lygia Stewart, UCSF & SF VAMC, United States; Emmanouil Giorgakis, University of Arkansas for Medical Sciences, United States; Philip Rosenthal, University of California, San Francisco, United States; David Kevin Imagawa, University of California Irvine Medical Center, United States; Paulo N. Martins, University of Massachusetts, United States; Charles Mahlon Vollmer, University of Pennsylvania, United States; Amer Zureikat, University of Pittsburgh Medical Center, United States; Jessica Lindemann, Washington University, United States; Philip Steven Barie, Weill Cornell Medicine, United States; Daniel Czarnevicz, SMI, Uruguay; Ruben David Hernandez, Hospital de Clínicas Caracas, Venezuela; Nawal Mohammed Al-Halli, Sana'a University, Al-Thawra Modern General Hospital, Yemen; Mohammed Mohammed Al-Shehari, Sana'a University, Al-Thawra Modern General Hospital, Yemen; Onesai Blessing Chihaka, Parirenyatwa Group of Hospitals, Zimbabwe.

**Country Leaders:**

**Algeria:** Kamel Bentabak, Pierre and Marie Curie Cancer Center, Algiers; Salah Eddine Kacimi, Faculty of Medicine, University of Tlemcen Tlemcen. **Australia:** Mehrdad Nikfarjam, University of Melbourne, Melbourne. **Belarus**: Aliaksei Shcherba, Minsk medical Center for Surgery, Transplantation and Hematology Minsk. **Belgium:** Gregory Sergeant, Jessa Ziekenhuis Hasselt. **Brazil:** Gustavo Coelho, Federal University of Ceara Fortaleza. Orlando Torres, Maranhão Federal University Sao Luis. **Bulgaria**: Nikolay Belev, UMHAT-"Eurohospital", Medical Training and Simulation Centre, MU-Plovdiv; Burundi Fabrice, Muhezagiro University of Burundi, Bujumbura. **Canada:** Ephraim Tang, London Health Sciences Centre - Western University London, Ontario; Janet Martin, Western University London. **Chile**: Christian Diaz, Hospital Dr Sotero del Rio, Santiago; Nicolas Devaud Instituto Oncologico Fundacion Arturo Lopez Perez (FALP) Santiago. **China**: Kongyuan Wei, Heidelberg University Hospital/ Chinese PLA General Hospital, Beijing; Maher Hendi, Zhejiang University School of Medicine, Sir Ren Ren Shaw Hospital, Hangzhou. **Croatia**: Danko Mikulic University Hospital Merkur Zagreb. **Cyprus**: Nikolaos Gouvas, University of Cyprus, Nicosia. **Czech Republic**: Andrej Nikov, University Hospital Kralovske Vinohrady, Prague. **Egypt**: Dalia Fathallah, Alexandria University Main Hospital, Alexandria; Mahmoud Saad, Assiut Faculty of Medicine, Assiut. **Estonia**: Olav Tammik, University Clinic of Tartu, Tartu. **Finland:** Heikki Huhta, Oulu University Hospital, Oulu. **France:** Laurent Sulpice, University Hospital Rennes, Rennes; Renato Lupinacci, Ambroise Pare Hospital, Paris-Saclay University, Paris. **Germany**: Gregor Stavrou, Klinikum Saarbrücken. **Greece** Evangelos Felekouras, National and Kapodistrian University of Athens; Vasileios Papaziogas, Aristotle University of Thessaloniki. **India**: Sanjeev Misra, All India Institute of Medical Sciences Jodhpur. **Indonesia**: Erik Prabowo Diponegoro University / Kariadi General Hospital, Semarang. **Iraq**: Hashim Talib Hashim University of Baghdad, College of Medicine, Baghdad; Maytham Al-Juaifari Al-Sader, Teaching Hospital, An Najaf. **Japan**: Sohei Satoi, Kansai Medical University Hirakata. **Jordan**: Khaled Obeidat, Jordan University of Science and Technology, Irbid; Maram Mohsen, Jordan University Hospital, Amman. Republic of **Korea,** Ho-Seong Han, Seoul National University Bundang Hospital, Seongnam. **Lebanon**: Mohamad Khalife, American University of Beirut. **Libya**: Muhammed Elhadi, Faculty of Medicine, University of Tripoli. **Lithuania**: Audrius Dulskas, National Cancer Institute Vilnius. **Malaysia**: Jin Bong Sunway Medical Center Petaling Jaya. **Maldives:** Shahi Ghani, Tree Top Hospital, Male'. Mexico: Alejandro Eduardo Padilla, Rosciano National Cáncer Institute, Mexico City; Javier Melchor-Ruan, National Cancer Institute Mexico. **Mongolia**: Sarnai Erdene, Mongolian National University of Medical Sciences, Ulaanbaatar. **Morocco**: Amine Benkabbou, National Institute of Oncology, Mohammed V University in Rabat. **Namibia**: Pueya Nashidengo, Windhoek Central Academic Hospital/University of Namibia School of Medicine, Windhoek. **New Zealand**: Jonathan Koea, North Shore Hospital, Auckland. **Nigeria** Ademola Adeyeye, Afe Babalola University Ado-Ekiti(ABUAD) Ado-Ekiti; Olusegun Alatise, Obafemi Awolowo University, Ile Ife. **Pakistan:** Sami Ullah Services Hospital Lahore Lahore. Palestine, Mustafa Abu Jayyab Alshifaa Hospital, Gaza; Sarah Amro, Alia Govermental Hospital, Hebron; Walaa Mohammed Alnammourah, Alia Governmental Hospital, Hebron. **Philippines**: Catherine The, Makati Medical Center, Makati. **Poland**: Michał Pędziwiatr, Jagiellonian University Medical College, Kraków; Wojciech Polkowski, Medical University of Lublin. **Romania**: Sorin Barbu, "Iuliu Hatieganu" University of Medicine & Pharmacy, Cluj-Napoca **Serbia**: Aleksandar Karamarkovic, Surgical Clinic "Nikola Spasic", University Clinical Center "Zvezdara", Belgrade, Faculty of Medicine University of Belgrade; Daniel Galun, Clinic for Digestive Surgery, Clinical Center of Serbia, Belgrade. **Singapore**: Brian Goh, Singapore General Hospital, Singapore. **Slovenia**: Blaž Trotovšek, University Medical Centre Ljubljana. **South** **Africa**: Jones Omoshoro-Jones, Chris Hani Baragwanath Academic Hospital/University of the Witwatersrand, Johannesburg. **Spain**: Benedetto Ielpo, Hospital del Mar, Barcelona. **Sudan**: Abdelfatah Abdelmageed, Omdurman Islamic University, Khartoum. **Sweden**: Per Sandström, University Hospital of Linkoping. **Switzerland**: Alessandra Cristaudi, Ospedale Regionale di Lugano; Beat Gloor, Inselspital, University Bern; Christoph Kuemmerli, Clarunis, University Centre for Gastrointestinal and Liver Diseases, St. Clara Hospital and University Hospital, Basel. **Syria**: Alaa Hamdan Tishreen, University Hospital Lattakia; Mohammad Karam Chaaban Al-Mouwasat University Hospital, Damascus. **Taiwan**: Chien Hui Wu, National Taiwan University Hospital, Taipei; Po-Chih Yang Fu Jen, Catholic University Hospital, New Taipei City. **Tunisia**: Ammar Houssem Sahloul Hospital, Sousse; Oussama Baraket, Hopital Habib Bouguetfa de Bizerte, University Tunis El Labar Bizerte. **Turkey**: Ahmet Çoker Ege University, Izmir HPB Clinic, Izmir. **United Kingdom**: Mark Taylor, Belfast Health and Social Care Trust, Belfast; Nigel Jamieson, University of Glasgow; Satheesh Iype, Royal Free Hospital London. **United States of America**: Emmanouil Giorgakis, UAMS Medical Center/ Winthrop P Rockefeller Cancer Institute, Little Rock; Motaz Qadan, Massachusetts General Hospital, Boston; Sabha Ganai, University of North Dakota, Sanford Health, Fargo. **Yemen**: Hamza Al-Naggar, Faculty of Medicine - Sana'a University, Sana'a. **Zimbabwe**: Onesai Chihaka, University of Zimbabwe, Harare.
