# Supplementary material for: Hospital-Level Determinants of Mortality After Pancreas Surgery: A Global Infrastructure Analysis
Source: Ann Surg. 2026 Sep 9;284(5):985–92. doi: 10.1097/SLA.0000000000007196 (PMC13652114; doi:10.1097/SLA.0000000000007196)
Supplement: Supplementary file 2 [file sla-284-0985-s002.docx]

| **Survey Question Number** | **Hospital Factor** | **Survey phrasing** |
| --- | --- | --- |
| 4b | Interventional radiology/endoscopy on-site availability | How important do you rate the availability of specialized units such as interventional radiology or advanced endoscopy (e.g. ERCP) at the hospital site where the pancreatic surgery was performed, in preventing FTR?  Availability at my institution: Yes/No |
| 4d | Equipment maintenance | How crucial do you rate the availability of hospital resources to address poorly maintained equipment or outdated facilities, potentially compromising highest standards patient care, with regards to FTR?  Availability at my institution: Yes/No |
| 7b | Evidence adoption | How important is the institution's openness to adopting newer, evidence-based practices into the routine management of patients undergoing pancreatic surgery, in influencing FTR? Availability at my institution: Yes/No |
| 8a | Structured nutrition support | How critical do you rate the administration of proper nutritional support and counselling after pancreatic surgery in preventing FTR in your institution?  Availability at my institution: Yes/No |
| 9a | Favorable nurse-patient ratios | How crucial do you rate the influence of nurse-to-patient ratios in specialized units to prevent FTR after pancreatic surgery in your institution? Availability at my institution: Yes/No |
| 9b | Systematic staff training | How significant would you rate a specialized training of staff and nurses in the management of post-pancreatectomy complications in influencing FTR outcomes? Availability at my institution: Yes/No |

**Supplementary Table 1. Survey questions and phrasing for the six Hospital Resource Index (HRI) components.** Each survey item first assessed the respondent's perception of factor importance, followed by a binary assessment of availability at their institution. Only the availability response was used in analysis.

| **Variable** | **OR** | **95% CI** | **p-value** |
| --- | --- | --- | --- |
| HRI (per point) | 0.88 | 0.80–0.97 | **0.013** |
| High volume (>55/year) | 0.66 | 0.49–0.89 | **0.006** |
| Low/Middle HDI | 1.21 | 0.69–2.12 | 0.516 |
| Age >65 years | 1.37 | 1.00–1.89 | 0.051 |
| Malignant indication | 0.76 | 0.49–1.18 | 0.216 |
| ASA ≥3 | 1.60 | 1.18–2.17 | **0.002** |
| Vascular resection | 1.08 | 0.72–1.63 | 0.717 |
| OR = odds ratio; CI = confidence interval; HRI = Hospital Resource Index; HDI = Human Development Index; ASA = American Society of Anesthesiologists physical status classification. Model adjusted for all variables shown with cluster-robust standard errors for hospital-level correlation (206 clusters). Left-sided pancreatectomy and enucleation excluded. | | | |

**Supplementary Table 2.** Sensitivity analysis: Multivariable predictors of 90-day mortality restricted to pancreatoduodenectomy and total pancreatectomy (n=2,115). This table confirms that HRI remains significant (OR 0.88, p=0.013) after excluding distal pancreatectomies and enucleations. Notably, volume is also significant in this subgroup (OR 0.66, p=0.006), which reinforces that *both* factors matter independently.

|  | **Low Volume (≤55/yr)** | **High Volume (>55/yr)** |
| --- | --- | --- |
| **HRI 5-6** | 9.7% mortality | 7.1% mortality |
| **HRI 0-4** | 12.0% mortality | 11.4% mortality |
| *HRI = Hospital Resource Index* | | |

**Supplementary Table 3. Ninety-day mortality by high and low HRI rates stratified by volume and HDI.** Formal interaction testing confirmed no significant interaction between HRI and volume (p=0.265), indicating additive rather than synergistic effects. These descriptive data complement the adjusted analysis and further support the independent contributions of both HRI and caseload volume.

| **HDI** | **HRI 0-4** | **HRI 5-6** | **p-value** |  |  |
| --- | --- | --- | --- | --- | --- |
| Low-Middle (n=119) | 14.8% mortality | 8.6% mortality | 0.299 |  |  |
| High (n=310) | 8.9% mortality | 9.2% mortality | 0.917 |  |  |
| Very High (n=2,499) | 12.1% mortality | 7.9% mortality | 0.001 |  |  |
| *HRI = Hospital Resource Index; HDI = Human Development Index* | | | | |  |

**Supplementary Table 4. Ninety-day mortality rates by HRI category, stratified by country Human Development Index.** P-values from chi-square tests. Formal interaction testing confirmed no significant HRI×HDI interaction (p=0.703), indicating the protective effect of HRI does not differ significantly across HDI strata; non-significant results in smaller subgroups likely reflect limited power.

| **Variable** | **OR** | **95% CI** | **p-value** |
| --- | --- | --- | --- |
| HRI (per point) | 0.93 | 0.87–0.99 | **0.033** |
| High volume (>55/year) | 0.99 | 0.80–1.23 | 0.932 |
| Low/Middle HDI | 1.27 | 0.76–2.10 | 0.358 |
| Age >65 years | 1.11 | 0.95–1.29 | 0.192 |
| Malignant indication | 0.94 | 0.79–1.12 | 0.477 |
| ASA ≥3 | 1.17 | 0.94–1.46 | 0.162 |
| Distal pancreatectomy | 0.75 | 0.64–0.89 | **0.001** |
| Vascular resection | 1.16 | 0.93–1.45 | 0.183 |
| *OR = odds ratio; CI = confidence interval; HRI = Hospital Resource Index; HDI = Human Development Index; ASA = American Society of Anesthesiologists physical status classification.* | | | |

**Supplementary Table 5. Multivariable predictors of major complications (Clavien-Dindo ≥3a).** Model adjusted for all variables shown with cluster-robust standard errors for hospital-level correlation, mirroring the mortality analysis. HRI was associated with reduced complications (p=0.033), while hospital volume showed no significant association (p=0.932), consistent with the failure-to-rescue hypothesis.
